# Temporal transcriptome analysis of the chicken embryo yolk sac

**DOI:** 10.1186/1471-2164-15-690

**Published:** 2014-08-19

**Authors:** Liran Yadgary, Eric A Wong, Zehava Uni

**Affiliations:** Department of Animal Science, The Robert H. Smith Faculty of Agriculture, Food and Environment, The Hebrew University, 76100 Rehovot, Israel; Department of Animal and Poultry Sciences, Virginia Tech, 24061 Blacksburg, VA USA

**Keywords:** Yolk sac, Gene expression, Transcriptome analysis, Chicken embryo, Epithelial cells, Nutrient transport, Lipoproteins, Cytoskeleton, Erythropoiesis

## Abstract

**Background:**

The yolk sac (YS) is an extra-embryonic tissue that surrounds the yolk and absorbs, digests and transports nutrients during incubation of the avian embryo as well as during early term mammalian embryonic development. Understanding YS functions and development may enhance the efficient transfer of nutrients and optimize embryo development. To identify temporal large-scale patterns of gene expression and gain insights into processes and mechanisms in the YS, we performed a transcriptome study of the YS of chick embryos on embryonic days (E) E13, E15, E17, E19, and E21 (hatch).

**Results:**

3547 genes exhibited a significantly changed expression across days. Clustering and functional annotation of these genes as well as histological sectioning of the YS revealed that we monitored two cell types: the epithelial cells and the erythropoietic cells of the YS. We observed a significant up-regulation of epithelial genes involved in lipid transport and metabolism between E13 and E19. YS epithelial cells expressed a vast array of lipoprotein receptors and fatty acid transporters. Several lysosomal genes (*CTSA*, *PSAP*, *NPC2*) and apolipoproteins genes (*apoA1*, *A2*, *B*, *C3*) were among the highest expressed, reflecting the intensive digestion and re-synthesis of lipoproteins in YS epithelial cells. Genes associated with cytoskeletal structure were down-regulated between E17 and E21 supporting histological evidence of a degradation of YS epithelial cells towards hatch.

Expression patterns of hemoglobin synthesis genes indicated a high erythropoietic capacity of the YS between E13 and E15, which decreased towards hatch. YS histological sections confirmed these results. We also observed that YS epithelial cells expressed high levels of genes coding for plasma carrier proteins (*ALB*, *AFP, LTF*, *TTR*), normally produced by the liver.

**Conclusions:**

Here we expand current knowledge on developmental, nutritional and molecular processes in the YS. We demonstrate that in the final week of chick embryonic development, the YS plays different roles to support or replace the functions of several organs that have not yet reached their full functional capacity. The YS has a similar functional role as the intestine in digestion and transport of nutrients, the liver in producing plasma carrier proteins and coagulation factors, and the bone marrow in synthesis of blood cells.

**Electronic supplementary material:**

The online version of this article (doi:10.1186/1471-2164-15-690) contains supplementary material, which is available to authorized users.

## Background

During embryonic development, nutrients are transferred to the embryo by specialized tissues. In mammals, a continuous transport of nutrients takes place from the mother to the embryo through the placenta, however in early stages of gastrulation the mammalian embryo is also dependent upon the transport of nutrients from the yolk through its surrounding tissue, the yolk sac (YS) [1]. Different from mammals, the YS of the avian embryo supports its development during the entire phase of embryogenesis [2]. The chick embryo is separated from the mother and therefore must derive all of its nutrients only from the contents of the egg. This closed environment provides an excellent model to study mechanisms by which nutrients are utilized through the YS.

In the first week of chick embryonic development, the YS begins to form from the developing gut of the embryo. A layer of ectoderm starts to spread over the surface of the yolk, ultimately covering it entirely. Next, a rapidly proliferating population of endodermal cells spreads and forms an epithelial layer between the yolk surface and the ectoderm. Finally, cells of mesodermal origin migrate between the endodermal and ectodermal layers and form a mesodermal layer, which differentiates into blood vessels, blood cells and connective tissue [3–5]. Up to E11, very small amounts of yolk nutrients are transferred to the embryo by the YS. From E11, after the YS has totally surrounded the yolk, the epithelial cells of the YS function as the mediators for the transport of nutrients from the contents of the yolk to the blood circulation of the developing embryo [6].

The transition from early embryogenesis to the second phase of incubation is characterized by prompt utilization of nutrients through the YS and a rapid growth of the embryo [7]. The nutrients are absorbed into YS epithelial cells from the yolk content by receptor-mediated endocytosis of lipoproteins [5, 8] and by transporters for peptides, amino acids, glucose, and minerals [9, 10]. Endocytosis of lipoproteins into the YS epithelial cells has been confirmed by electron microscope observations of the YS [11], however out of the vast variety of receptors that mediate lipid entrance to endodermal cells, only two receptors for lipoproteins [5, 8] and no receptors for free fatty acids have been examined so far in the YS.

Inside YS epithelial cells, there is evidence of intensive lysosomal digestion of lipoproteins such as very low density lipoproteins (VLDL) into free fatty acids and glycerol, which are then reesterified to form triglycerides and phospholipids, and assembled into new lipoprotein particles (low density lipoproteins, LDL and high density lipoproteins, HDL) [5, 12]. The subsequent lipoproteins are exocytosed to the blood and transferred to the embryonic tissues [12–14]. Although the idea that a substantial lipid remodeling takes place within YS epithelial cells is supported by several studies, only a fraction of the genes involved in these processes have been examined in the YS. Furthermore, despite recent data on the capacity of the YS to express genes related to protein and carbohydrates synthesis, storage, digestion and transport [10, 15, 16], the developmental profiles of cellular and molecular mechanisms responsible for a larger part of the above mentioned processes have not been investigated in the YS.

The utilization of nutrients by the chick embryo, at any stage during development, has a direct effect on growth and hatchability. A more complete and large-scale picture of the processes and mechanisms that control yolk utilization through the YS is needed in order to better understand how avian and early term mammalian embryos utilize yolk nutrients. Therefore, the aim of the current study was to generate and analyze high-throughput gene expression data of the YS during the last week of incubation. Thus, we performed a transcriptome analysis of RNA from YS tissues, as well as histological and biochemical analyses, on days 13, 15, 17, 19, and 21 (day of hatch) of incubation. Our time-series experimental design, with 3 biological replicates per day, allowed us to quantify differential gene expression between days, identify temporal patterns of biological processes, and gain insights into the processes and mechanisms of nutrient synthesis, absorption, digestion, and secretion within the YS.

## Methods

### Eggs and yolk sac sampling

Fertile eggs were obtained from a commercial broiler breeder farm (Brown, Hod Hasharon, Israel). Eggs were incubated in a Petersime hatchery at the Faculty of Agriculture of the Hebrew University under standard conditions (37.5°C, 60% relative humidity, with automatic egg turning). Six eggs, representing the weight distribution of the eggs at set, were selected at each of the following days: E13, E15, E17, E19, and E21 (day of hatch; no later than 30 min after hatch). Embryos were killed by cervical dislocation. The experiment was approved by the Ethics Committee for Animal Experimentation, Faculty of Agricultural, Food and Environmental Sciences, The Hebrew University of Jerusalem. The YS was separated from the yolk and weighed, and a small piece from the area vasculosa, where endodermal epithelial cells and villus-like folds develop and which is the vascularized and most active region in endocytosis of yolk, was rinsed in a 0.9% autoclaved saline solution, and placed in microcentrifuge tubes at -80°C for mRNA analysis. Fractions of YS were also taken for histological sectioning.

Six additional eggs were sampled on each examined day. Embryos were weighed, the yolk + YS were weighed, and the yolk + YS were homogenized and stored at -20°C for total fat content and fatty acid analyses. In the current study we present quantities of lipids in the yolk + YS because according to Yadgary et al. [7], estimating utilization of nutrients by the embryo is more accurate if calculated from the yolk + YS, and not just from the yolk.

### Total RNA isolation and pooling of samples

Libraries for next generation sequencing (NGS) were prepared from total YS RNA. Total RNA was isolated from 30 YS (six replicates per day) using TRI-Reagent RNA/DNA/Protein Isolation Reagent 5 according to the manufacturer’s protocol (Sigma-Aldrich, St. Louis, MO). 50 to 100 mg of tissue was homogenized with 1 mL of TRI-Reagent and phase separated using chloroform. RNA was precipitated using isopropanol, washed with 75% ethanol, and solubilized with nuclease-free water. RNA concentration was determined spectrophotometrically in a Nanodrop ND-1000 (NanoDrop, Wilmington, DE). Equal amounts of YS RNA were pooled from two replicate embryos so that a total of 15 libraries were prepared for NGS- three biological replicates per each examined day. Prior to sequencing, all samples were treated with DNase and quality assessed on a Bioanalyzer 2100 (Agilent, Santa Clara, CA)*.*

High-throughput Serial Analysis of Gene Expression (SAGE) was performed using SOLiD4 technology (Applied Biosystems) at The Center for Genomic Technologies at the Hebrew University of Jerusalem. RNAs were reverse transcribed and SAGE libraries were prepared using a SOLiD SAGE Kit with Barcoding Adaptor Module (Applied Biosystems) which allows multiple samples to be processed in parallel. The system generates a library of 27-bp “tags” for all the transcripts in a cell and provides a method for analyzing the genome-wide expression levels of poly(A) transcripts. Briefly, mRNAs were attached to poly(T) coupled magnetic beads, cDNAs were synthesized using SuperScriptR III Reverse Transcriptase and *E. coli* DNA polymerase, digested with a 4-bp cutter enzyme (NlaIII), and ligated to a barcode adaptor sequence (unique for each library) containing a recognition site for a restriction enzyme (EcoP15I) that cuts DNA 27 bp away. After restriction and separation from beads, the tags were ligated to a second adaptor. The 27-bp transcripts, surrounded by two adaptors, were pooled into a single multiplexed sequencing reaction. Applied Biosystems SOLiD sequencer generated color-space fasta files (.csfasta) and the corresponding quality control files (.qual) for 14 out of the 15 libraries. Due to a technical error, one sample from E19 was not sequenced. All csfasta and quality data sets supporting the results of this article are available in the SRA repository (http://www.ncbi.nlm.nih.gov/Traces/sra/sra.cgi?study=SRP045315) with accession number SRP045315.

A total of 179,000,718 sequencing reads were obtained (Additional file 1). All the reads (50 bp long) were subjected to adaptor sequence trimming and quality filtering using the FastX-toolkit suite and FASTQ manipulation tool [17] available through the Web-based bioinformatics platform Galaxy (http://usegalaxy.org/). Based on the FastX quality statistics, all the reads were trimmed to 23-bp and low phred scored reads were discarded. One sample from E21 showed consistently low phred scores and therefore was excluded from further analyses. The remaining reads obtained from each library (on average ~80%) were aligned against the chicken transcriptome RefSeq (17,696 mRNA Ref-Seq, downloaded from NCBI database). Because the SAGE protocol is designed to produce reads that represent mRNA regions adjecent to the 3'-most NlaIII endonuclease cleavage site, we mapped reads only to 15,169 Ref-Seq mRNA sequences that were computationally found to be longer than 22 bases after restriction enzyme cleavage and were non-redundant in the 23-bp mRNA region, to which the reads were predicted to be aligned. Sequence mapping was done using bowtie software [18] in a Galaxy setting, with maximum number of mismatches permitted [n] = 2, maximum permitted total of quality values at mismatched read positions (-e) = 70, and maximum alignments authorized [m] = 10. Reported alignments were chosen to be best in criteria [n] and criteria (-e) (--best). On average, ~55% of the sequences of all libraries were mapped. Of the mapped reads, on average 93% were unique, i.e. mapped to only one Ref-Seq mRNA sequence.

The number of reads that were mapped to each gene was counted and count data was then used as input for JMP GENOMICS 6.0 (SAS, Cary, NC) software analyses. Read counts of the libraries were normalized using the Upper-Quartile Normalization method [19], and were then log_e_ (ln) transformed, yielding transformed normalized counts. Principal component analysis (PCA) was performed to identify outliers within each day (Additional file 2). One outlier was detected on E17 and discarded, leaving 12 samples (three replicates on E13 and E15, and two replicates on E17, E19, and E21) for further analyses. Outlier detection was confirmed by manual inspection of data from 200 random genes (Additional file 2). A generalized linear model (GLM) based on the negative binomial distribution for count data was performed to test for differential expression between embryonic days (JMP GENOMICS 6.0, SAS, Cary, NC). Genes that were found to have a low normalized count number across incubation (average <8) were not included in the GLM. Thus, a total of 7128 genes were tested at significance level of α = 0.05 adjusted to match a 5% false discovery rate [20]. It should be noted that even though throughout the manuscript we use the term "expressed" for genes with ≥8 normalized mean count across samples, it would be incorrect to claim that the filtered genes are not expressed in the YS; It is possible that a higher coverage (sequencing depth) would detect genes with very low copy number that were not detected in our experiment.

For each one of the 7128 genes, mean count number (transformed normalized) was calculated for each embryonic day. To examine temporal changes during incubation, per each day the mean count number of each gene was standardized to the standard deviation of all the values of the genes. Significantly changed genes (3547) were then subjected to hierarchical clustering (JMP GENOMICS 6.0, SAS, Cary, NC). Genes clustered together were annotated using the web-based Database for Annotation, Visualization, and Integrated Discovery (DAVID) [21].

### Lipids analyses

**Total Lipid Analysis.** Total lipids were extracted using a modified method of Folch et al. [22], as previously described by Yadgary et al., [7]. Briefly: 10 mL of chloroform-methanol (2:1 vol/vol) was added to 0.5 mL of YS homogenates. After 30 min, 2 mL of distilled water was added. Following overnight incubation at 4°C, 5 mL of the bottom layer was transferred to preweighed, glass tubes and oven-dried at 105°C. The tubes with the remaining fat were weighed and the weight of fat per gram of YS + yolk and per total fat in the YS + yolk was calculated.

**Fatty acid analysis.** Fatty-acid concentration was determined in lyophilized samples of 100 μg YS + yolk by gas chromatography on a DEGS column. Absolute amounts of lipids were determined after addition of an internal standard of heptadecanoic acid, and the proportion of specific fatty acid was calculated out of the total fatty-acid concentration. The concentrations of saturated fatty acids and unsaturated fatty acids were determined.

### Histology

Fresh sections of YS were fixed overnight in 4% (v/v) buffered formaldehyde. Serial 4-μm sections were prepared after the samples had been dehydrated in graded ethanol solutions, cleared in chloroform and embedded in paraffin. Sections were deparaffinized in xylene, rehydrated, stained with hematoxylin and eosin or with proliferating cell nuclear antigen (PCNA) immunostaining and evaluated by light microscopy.

## Results and discussion

### Mapping and statistics

Serial Analysis of Gene Expression (SAGE) methodology followed by NGS was performed on 2–3 biological replicates of mRNA extracted from YS on E13, E15, E17, E19 and E21. Using what is also known as SAGE-Seq, a single region of each mRNA molecule was sequenced, and thus the number of reads mapped to a specific mRNA reference sequence was directly related to expression levels of that gene. Reads were mapped using 15,169 chicken mRNA Ref-Seq, however only 7,128 genes were further analyzed after excluding genes with very low count number. For each of the 7,128 genes, after normalization and logarithmic transformation of the data, we identified differential expression across days, and calculated mean count number per embryonic day (i.e. ln normalized expression).

### Clustering and functional annotation

**Figure 1:**
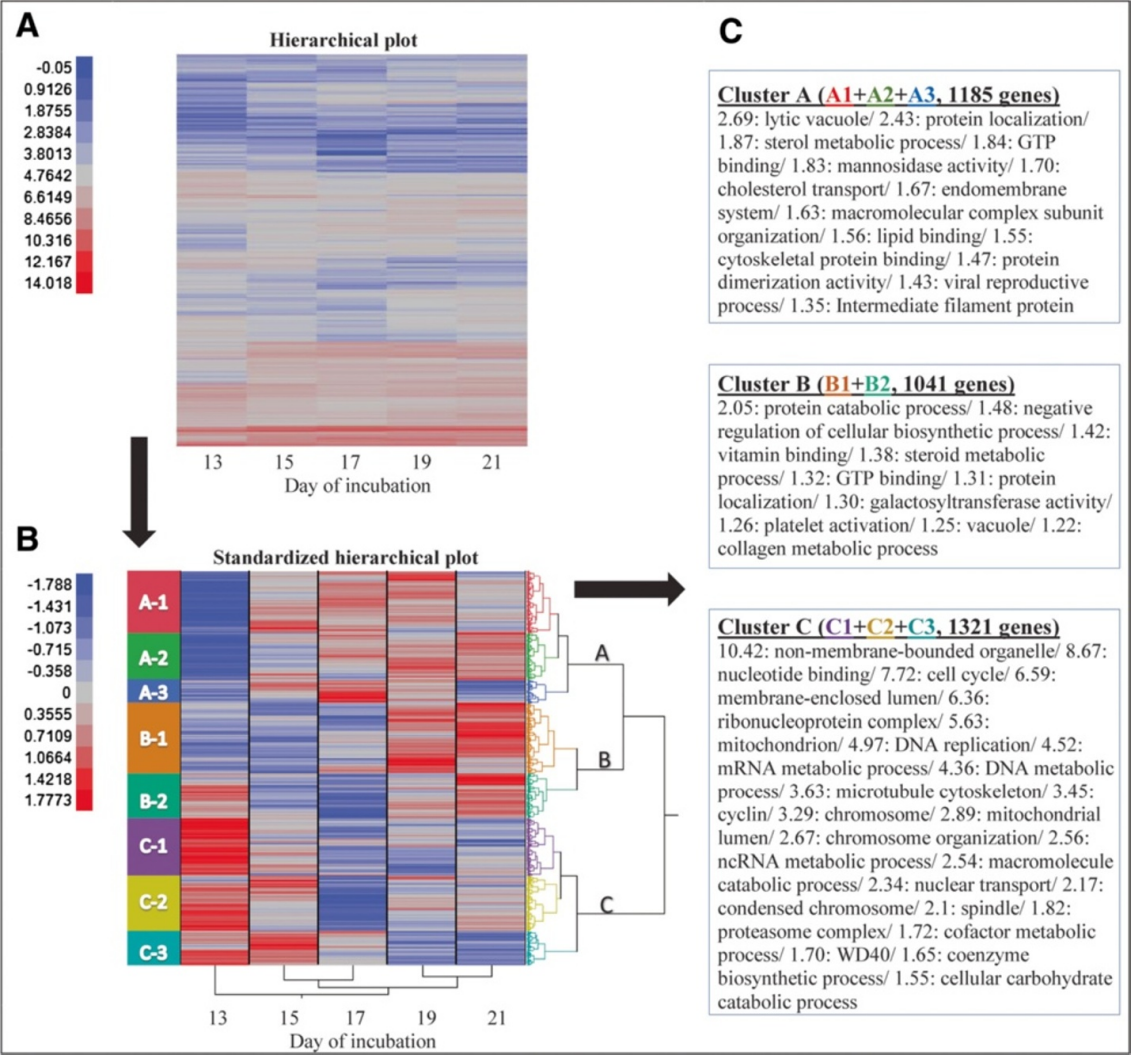
**Time-course hierarchical clustering and functional annotation of the 3,547 genes that exhibited a significant change in expression levels in the YS during incubation. A**. Heatmap showing the expression of the genes before standardization of the data. A blue-red color palette illustrates the log_e_ gene expression values, with blue corresponding to low and red corresponding to high expression. Clustering of these data is based firstly on the expression levels of the genes and within these levels on expression patterns. **B**. Heatmap showing the expression of the genes after standardization of the data. A blue-red color palette illustrates the standardized values, with blue corresponding to low and red corresponding to high expression. Cluster analysis was performed on the genes according to their temporal expression profiles and the corresponding dendrogram is shown to the right of the heatmap. The letters on the dendrogram **(A, B** and **C)** refer to the 3 major patterns of expression as described in results and discussion. The major 3 clusters were further divided (A1, A2, A3; B1, B2; C1, C2, C3) and colored, as shown to the left of the heatmap. C. DAVID annotation analysis for genes of the 3 major clusters. DAVID enrichment scores were assigned for each functional annotation group.

3,547 genes exhibited a significantly changed expression across days (p < 0.05, after FDR correction). To examine time-course expression patterns, we performed hierarchical clustering analyses to all significant genes after standardization of the data. Figure 1A shows the heat map for hierarchical clustering of the expression levels of the genes between E13 and E21 before we standardized the data. Clustering of these data would be based firstly on the expression levels of the genes and within these levels on expression patterns. However using standardization, we sought to cluster genes together on the basis of their temporal expression patterns, regardless of their specific values, as shown in Figure 1B.

**Figure 2:**
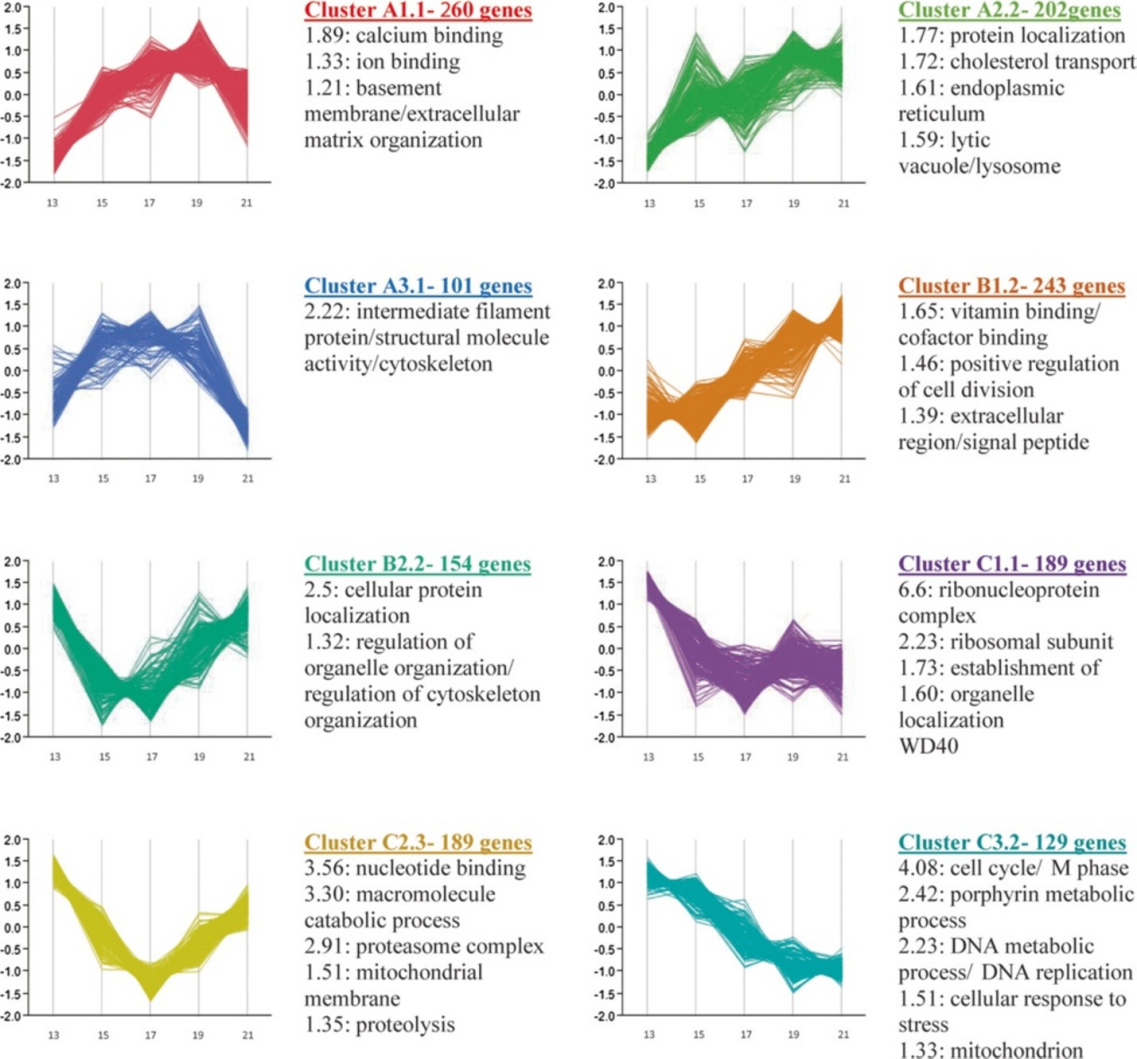
**Further partitioning and functional annotation analysis to genes clustered together according to their temporal expression pattern.** DAVID annotation analysis and enrichment scores for 8 selected clusters (out of 21) and a graphical representation of their gene expression. The full-version figure with functional annotation analysis and graphics for all 21 clusters is available as Additional file 3.

Different levels of partitioning into clusters demonstrated several distinct temporal patterns of change (Figures 1B, 2, Additional file 3). To explore common dynamic cell processes of these expression patterns, we performed a functional annotation analysis to genes clustered together at several levels of partitioning- we annotated the first 3 clusters (Figure 1C), the first 8 clusters (Additional file 3, gray background), and finally we annotated 21 further partitioned clusters that proved to add additional information on biological processes to the annotation data of the former clusters (Additional file 3, white background). To highlight the dynamics of expression changes and better visualize results, the final 21 clusters are presented as a continuous representation of gene expression (Additional file 3). A subset of 8 out of the 21 clusters are shown in Figure 2.

According to functional annotation, genes of cluster A, which exhibited a common pattern of increase in expression from E13 to E15 (Figure 1), were involved in several metabolic processes and pathways, most of them associated with lipid metabolism and protein transport. Partitioning of cluster A genes revealed that genes that were up-regulated from E13 to E19 and then down regulated until E21 were found as related to ion transport and extracellular matrix organization (cluster A1.1, Figure 2), genes that were up-regulated from E13 to E21 were associated with lipid and protein transport (cluster A2.2, Figure 2; cluster A2.1, Additional file 3), and genes that were up-regulated between E13 and E17 and then down-regulated until E21, were associated with proteolysis (Additional file 3) and structural actin cytoskeleton cell activity (cluster A3.1, Figure 2).

Genes of cluster B, which exhibited a common pattern of increase in expression from E17 to E19 (Figure 1), were enriched in GO terms and pathways that included, among others, protein catabolic process, negative regulation of cellular biosynthetic process, vitamin binding, and steroid metabolic process. Subdivision of these genes revealed that genes with no change in expression between E13 and E15 and then an up-regulation from E15 were involved in vitamin binding and in golgi activity such as sorting and packaging of proteins for transport and modification of oligosaccharides of glycoproteins (cluster B1.2, Figure 2; cluster B1, Additional file 3); genes that were down-regulated between E13 to E15 and then up-regulated from E17 to E21, were associated with protein transport to or within the endoplasmic reticulum (cluster B2.2, Figure 2; clusters B2.1, B2.2, B2.3, Additional file 3).

Genes of cluster C had a distinctly different temporal pattern of change compared to clusters A and B, as was observed in Figure 1B heat map and dendrogram. Cluster C1 genes exhibited a down-regulation between E13 and E17 in ribosomal biosynthesis processes, while cluster C2 genes were more associated with proteasome mediated protein breakdown and chromatin organization (Figure 2; Additional file 3). Furthermore, cluster C3 genes that were found to be involved in cell replication events and segregation of genetic material, exhibited a striking down-regulation between E15 and E21 (Figure 2, Additional file 3). Thus, expression of most of the genes in cluster C was indicative of a substantial reduction in YS cell proliferation.

### Synthesis of blood cells in yolk sac blood islands

**Figure 3:**
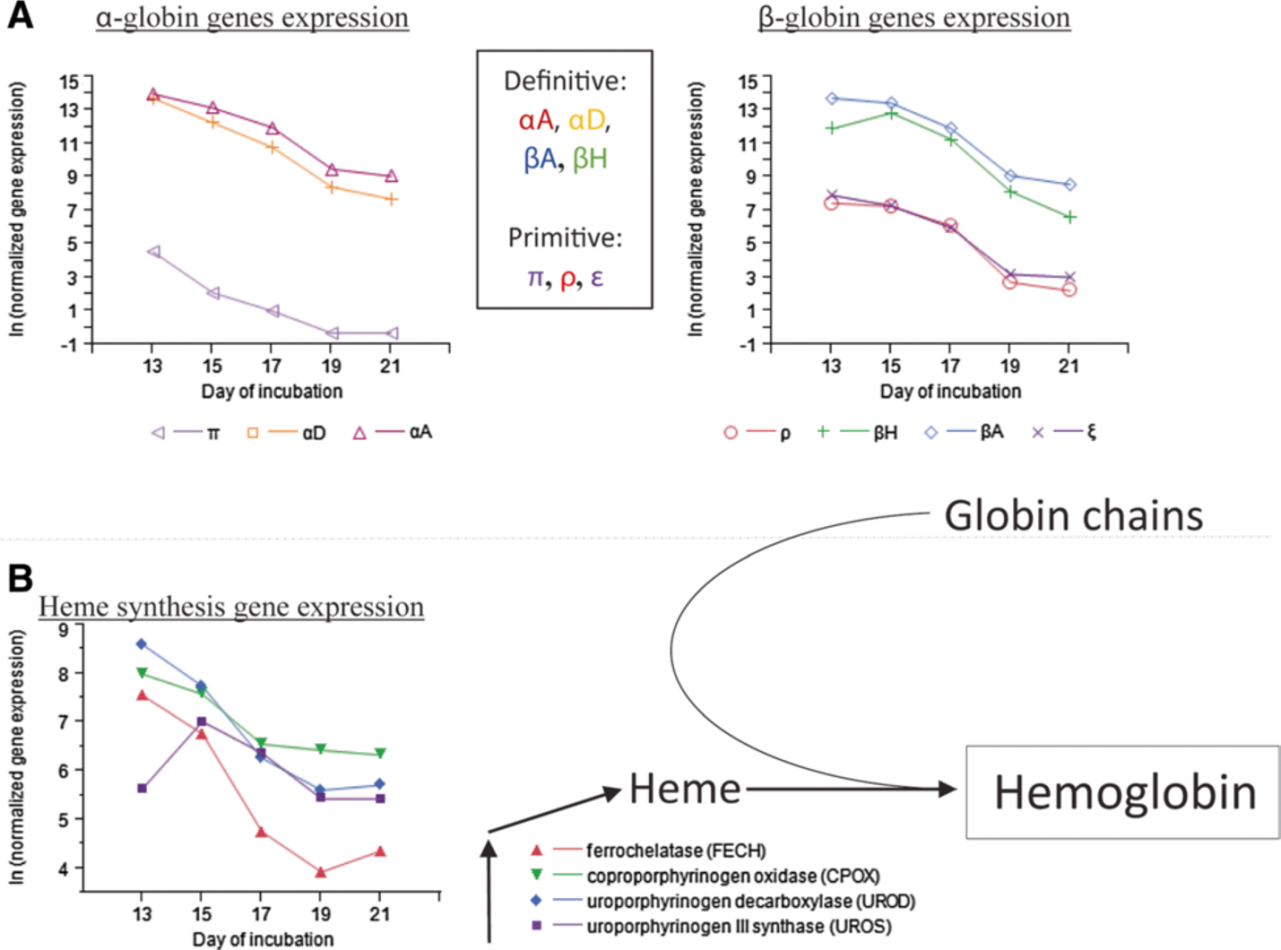
**Hemoglobin synthesis in the YS of the chick embryo during the last week of incubation. A**. Expression patterns of α-globin and β-globin genes in the YS. **B**. Expression patterns of heme synthesis genes in the YS.

**Figure 4:**
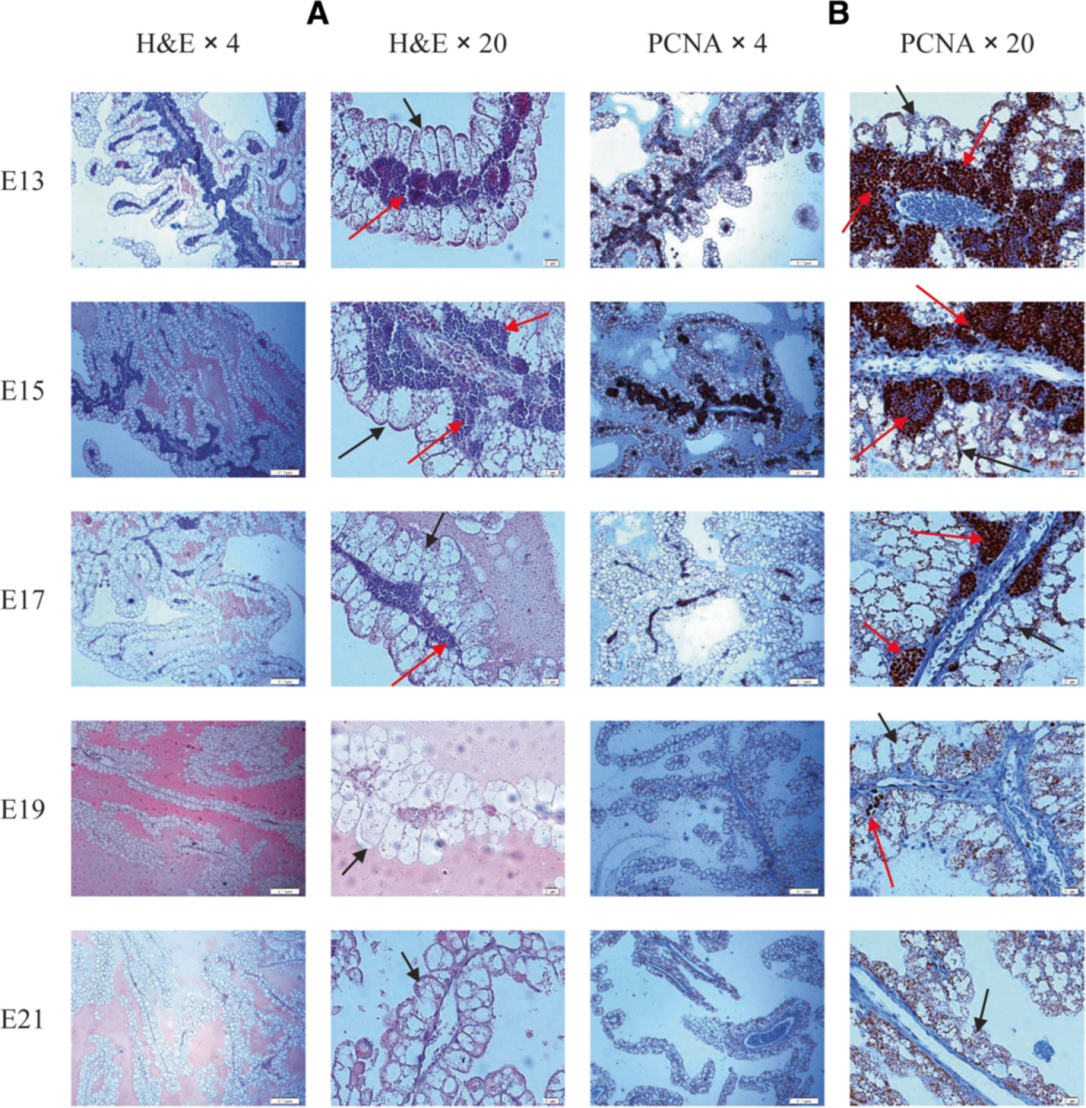
**Histology sections of the YS during the last week of chick embryonic development. A**. H&E stained YS sections on days E13 to E21. **B**. PCNA immunostained YS sections on days E13 to E21. Black arrows: Endodermal epithelial cells. Red arrows: Blood islands.

Clustering and functional annotation indicated that expression of YS genes involved in lipid metabolism and transport increased from E13 to E21, whereas genes involved in cell cycle and mitosis were substantially down regulated during the same period. Interestingly, we observed that the genes involved in cell replication were clustered together with genes involved in porphyrin metabolic process, i.e. hemoglobin synthesis (cluster C3.2, Figure 2). To elucidate the biological meaning of these results, we further examined the expression of genes involved in hemoglobin synthesis (Figure 3), and histologically monitored YS during the last week of embryonic development (Figure 4).

Hemoglobin is made up of globin chains and the porphyrin ring, or heme. The chicken globin genes include the α-globin gene cluster (π, αA, and αD ) and the β-globin gene cluster (ρ, βH, βA, and ϵ). Primitive erythrocytes in chickens express the π, ρ and ϵ globin chains, while definitive erythrocytes express αA, αD, βH, and βA [23, 24]. In the current study, we found that all seven globin genes were expressed in the YS, with a similar temporal pattern of down-regulation between E15 and E19 (Figure 3A). Significantly higher expression levels were observed for the definitive globins as compared to the primitive globins. Surprisingly, on E13 and E15 the definitive globins also exhibited the highest expression levels compared to all other chicken mRNA examined in the current study (Additional file 4).

The YS is considered as the sole niche for primitive erythropoiesis from E1 to E5, and as the main niche for early definitive erythropoiesis from E5 to E10 of chicken development [25]. Still, it is not clear how long the YS continues its erythropoietic process. Our results of the expression pattern of globin genes (Figure 3A), together with the similar pattern in heme synthesis gene expression (Figure 3B), indicated a very high erythropoietic capacity of the YS on E13 and E15, which decreases towards hatch. Our histological sectioning of the YS confirmed these results; we observed that blood islands, which produce blood cells in the YS, are abundant in the YS on E13 and E15, and then dramatically decrease towards hatch (Figure 4A). Moreover, using PCNA staining we observed that the cells in the blood islands were highly proliferative (Figure 4B). This explains the similar temporal pattern of hemoglobin genes and mitosis genes. Highly proliferative blood island cells produce vast amounts of hemoglobin on E13 and E15 and then lose contact from each other and from the endothelium, and then become free circulating cells. Thus expression of mitosis and hemoglobin genes substantially decrease towards hatch.

A transition is known to occur during late chick embryonic development, when the bone marrow becomes the major erythropoietic organ. There is however a controversy regarding the contribution of the YS to definitive erythropoiesis in the last week of chick embryonic development. While Nagai and Sheng [26] speculated that the YS continues to generate definitive erythrocytes at least until E15, Lassila et al. [27] suggested that from E11 erythropoiesis in the YS contributes less than 20% to peripheral blood cells. Our current study demonstrates that the YS is an erythropoietic tissue not only in the first week of incubation but also in the last week of incubation, with a massive migration of definitive red blood cells towards hatch. We therefore postulate that between E13 and E17 the YS contributes a greater part than assumed to the synthesis of red blood cells of embryos.

Our results represent the first quantitative look at YS erythropoiesis, and are in agreement with Niimi *et al.*[28] and Bruns and Ingram [23] who suggested that YS erythropoiesis lasts throughout incubation. However, in chick/quail chimaera studies, in which a quail embryo is grafted onto the YS of a chick very early in development, the majority of circulating blood cells in the last week of incubation were of the embryonic quail origin [29, 30]. The discrepancy between our results and those of chick/quail chimaeras studies can be resolved by considering similar experiments that found that from E7 the YS was extensively seeded by quail embryonic hematopoietic stem cells [31, 32]. Therefore during the last week of incubation, most of the proliferating cells in YS blood islands are of embryonic origin, making it impossible to differentiate between late-term chick YS and quail embryo blood cells in the chimaera studies.

### Epithelial cell gene expression analyses and annotation

Histology sectioning and annotational clustering revealed that in our temporal gene expression analyses we monitored at least two cell types- the endodermal epithelial cells and the erythropoietic cells of the YS. The YS also contains fibroblasts, endothelial cells, and vascular smooth muscle cells, and it is possible that at a low level we also sequenced mRNA from these cell types. However, we estimate that most of the sampled non-erythropoietic mRNA is from epithelial cells. Therefore, in the following sections, non-erythropoietic cells are referred to as YS epithelial cells.

Because the erythropoietic cells decreased in number, distinct down-regulation patterns of gene expression were observed in the YS (Figures 1 and 2). Expression patterns representing different cell types are not uncommon in high-throughput developmental time courses [33], however a more accurate representation of these patterns may be needed due to uneven numbers of the different cell types. Therefore, in the current study we fine-tuned gene expression analyses by computationally separating and normalizing genes associated with YS epithelium according to clustering analyses and GO terms (Additional file 5). Next, for each of the 3500 selected genes, we examined 4 contrasts between days of development; genes that were significantly up or down-regulated between E13 and E17, E17 and E21, E15 and E19, and E19 and E21 (P < 0.1, after FDR correction) were functionally annotated (Table 1). The normalized mean and P-value of the significant genes from each of the 4 contrasts are presented in Additional file 6. In addition, out of the selected 3,500 genes, the 50 highest expressed genes per each embryonic day, as well as the top 50 down-regulated and up-regulated genes during the examined period, are presented in Additional files 7 and 8, respectively.

**Table 1:** **Functional annotation of significantly down and up-regulated genes (Separate analysis for 3500 YS epithelial genes)**

Contrast (period)		Down regulated genes	Up regulated genes
13-17	n	348	458
		1.56: endoplasmic reticulum; 1.54 plasma membrane organization; 1.44: ubiquitin ligase complex; 1.33: purine nucleotide binding;	2.59: macromolecular complex subunit organization; 2.16: cholesterol metabolic process; 1.65: chaperone mediated protein folding requiring cofactor; 1.51: cholesterol transport; 1.3: vacuole;
17-21	n	391	256
		2.66: cytoskeletal part; 1.84: Intermediate filament protein; 1.74: actomyosin; 1.50: protein heterooligomerization; 1.25: lysosome	1.93: fatty acid metabolic process; 1.53: topological domain:Lumenal; 1.21: vitamin binding
15-19	n	269	389
		2.53: macromolecule catabolic process; 2.46: GTP binding; 1.61: nucleotide binding; 1.56: zinc ion binding; 1.27: establishment of protein localization	2.24: sterol metabolic process; 1.76: protein dimerization activity; 1.74: extracellular region; 1.59: response to wounding; 1.43: lipid oxidation; 1.34: transmembrane receptor protein serine/threonine kinase signaling pathway; 1.28: growth factor binding; 1.22: Collagen triple helix repeat; 1.20: Fibrinogen
19-21	n	191	110
		1.99: oxidation reduction; 1.59: keratin; 1.55: cholesterol metabolic process; 1.47: cofactor binding;	1.34: ossification; 1.25 membrane coat; 1.20: transmembrane region

DAVID enrichment scores were assigned for each functional annotation group.

The normalized mean and P-value of the significant genes from each of the 4 contrasts are presented in Additional file 6.

### Nutrient transport through yolk sac epithelium

**Figure 5:**
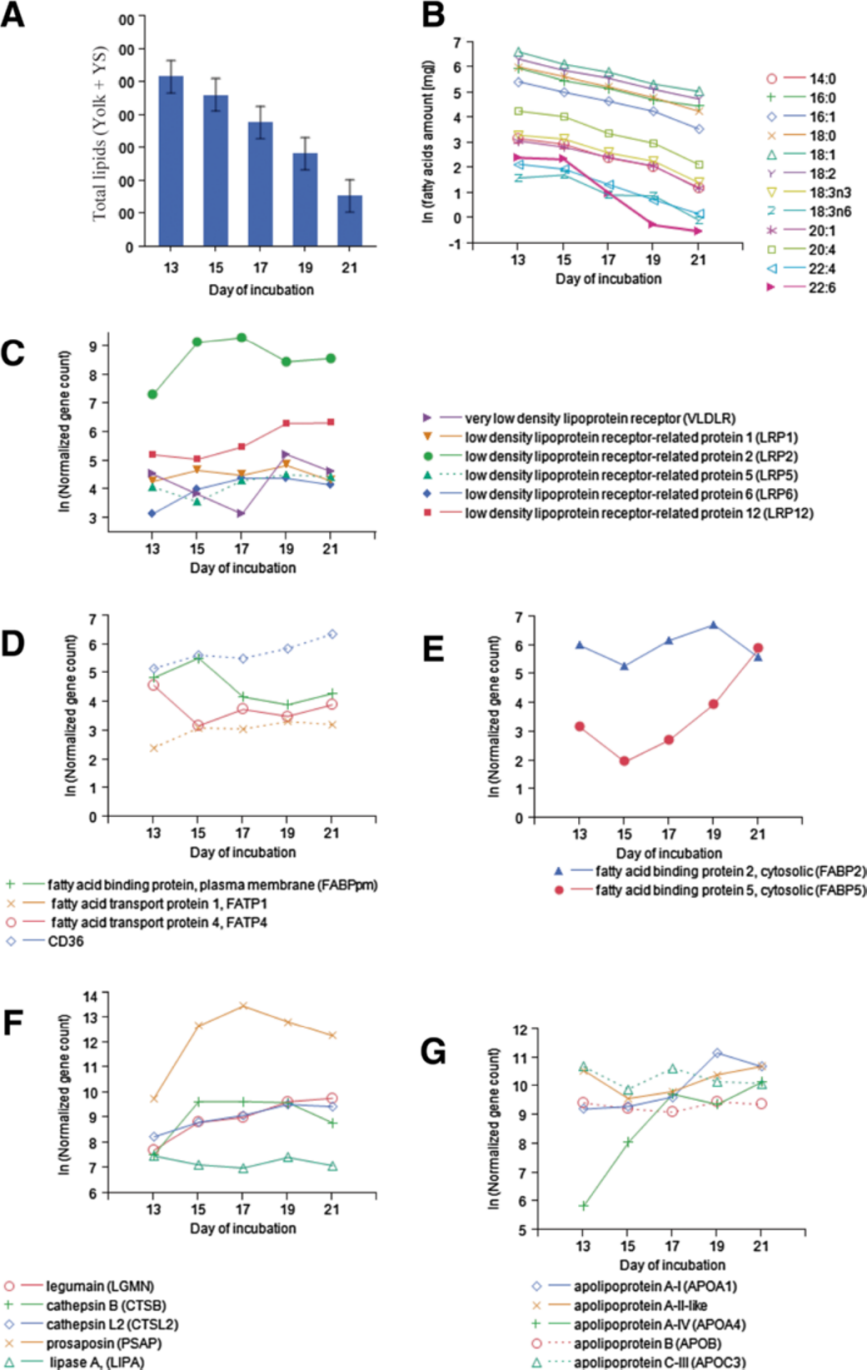
**Yolk Sac fat absorption and digestion during the last week of chick embryonic development. A**. Yolk + YS total fat content (mg). **B**. Yolk + YS fatty acids amount (log_e_ transformed). Gene expression levels of: **C**. lipoprotein receptors, **D**. membrane fatty acid transporters, **E**. fatty acid binding proteins, **F**. the 5 top expressed lysosome associated genes, and **G**. apolipoproteins in the YS.

YS epithelial cells are the mediators through which yolk nutrients are transferred to the blood stream of the embryo. Inside these cells, nutrients are digested and transformed by various processes [2]. In agreement with previous studies [6, 7, 9], in the current study we observed that yolk fat was extensively utilized through the YS by the embryo in the last week of incubation (Figure 5A). We examined the mechanisms by which yolk lipids are transferred to the embryo by analyzing the expression of YS epithelial genes. Contrasts and functional annotation showed a significant up regulation of genes involved in cholesterol transport and metabolic process between E13 and E17 as well as between E15 and E19 (Table 1). Processes further discussed include the endocytosis of lipoproteins, their digestion in the lysosomes of the epithelium, and the resynthsis and secretion of new lipoproteins.

The absorption of yolk lipids into YS epithelium is carried out by endocytosis of lipoproteins [11]. We found that a vast array of lipoprotein receptors mediate lipoprotein entrance to YS epithelial cells (Figure 5C). Among these receptors is VLDLR, which has a binding domain that recognizes VLDL and vitellogenin. VLDLR has been previously suggested to have a major role in YS lipoprotein uptake [8], however we show that *VLDLR* had relatively low levels of expression in the YS (Figure 5C). On the other hand LRP2, a receptor for a range of lipoproteins [34], had the highest expression level among all lipoprotein receptors. The high *LRP2* gene expression level increased from E13 to E17 and decreased from E17 to E21, supporting previous results of a study by Bauer et al. [5]. Expression levels of several lipoprotein receptors in YS epithelium (*LRP1*, *5*, *6*, *12*) may imply that *LRP2* does not have an exclusive role in YS lipoprotein uptake, however the putative genes *LRP5*, *LRP6*, and *LRP12* have not been shown to play a key role in lipoprotein clearance. Further research into gene expression levels as well as protein levels of lipoprotein receptors in the YS is needed to validate our results and to elucidate the contribution of each receptor to fat uptake through the YS.

Except for endocytosis of lipoproteins, other possible mechanisms of lipid uptake through YS epithelium, such as transporters for free fatty acids, have not been investigated. Previous studies have speculated that long chain poly-unsaturated fatty acids (22:6, 20:4) are differentially utilized from the yolk [35]. In the current study, we observed that between E15 and E19, docosahexaenoic (22:6) fatty acid, which is crucial for normal brain development, was more rapidly utilized from the yolk as compared to all other examined fatty acids (Figure 5B). To elucidate the differential utilization of yolk fatty acids, we examined the expression of genes responsible for absorption and transport of free fatty acids. Different membrane fatty acid transporters were expressed in YS epithelium (Figure 5D), as well as cytosolic fatty acid binding proteins that also play a role in uptake of long chain poly-unsaturated fatty acids (Figure 5E). Among them was *FABP5* which exhibited a substantial up-regulation between E15 and E21. Further research is needed to confirm the role of these transporters in the differential utilization of yolk fatty acids.

Inside YS epithelial cells, lipoproteins are transferred to lysosomes for digestion [8, 13, 14]. Yolk fat utilization is affected by the digestive capacity of these lysosomes. In the current study, several lysosomal digestion related genes were found to be among the 50 most highly expressed genes in YS epithelial cells (Additional file 7); Prosaposin, which facilitates the catabolism of glycosphingolipids in lysosomes, was the highest expressed gene from E15 to E21 (Figure 5F). We also found a high and constant expression of lipase A, which catalyzes the hydrolysis of cholesteryl esters and triglycerides in lysosomes (Figure 5F). In addition, many lysosomal proteases such as cathepsin A and B were highly expressed in the YS (Figure 5F). These results lend support to the hypothesis that yolk lipoproteins are hydrolyzed in the lysosomes of YS epithelial cells into free fatty acids, partial glycerides and glycerol.

Lipids are transported from the epithelial cells of the YS to the embryo as newly formed lipoproteins [12–14]. An integral part of these newly synthesized lipoproteins are apolipoproteins that serve as structural proteins and cell surface receptors. In the current work, apolipoprotein genes (*apoA1*, *A4*, *B*, *C3*, and the putative apo A-II-like) were among the most highly abundant in the YS (Additional file 7). We observed that expression of *apoB* and *apoC3* was constant, whereas *apoA4* exhibited a significant up-regulation between E13 and E17, while *apoA1* was up-regulated between E17 and E19 (Figure 5G). Our results reflect the intensive re-synthesis of lipoproteins in the YS and indicate that both HDL and LDL are produced in the YS and are then secreted to the embryonic circulation as previously suggested by Kanai et al. [12].

In summary, absorption, digestion and transport of lipoproteins are perhaps the main mechanisms by which yolk nutrients are utilized through YS epithelial cells. However, previous studies [10, 15], which examined YS expression of a small number of solute carrier (SLC) genes, suggested that they may play an important part in the uptake of peptides, amino acids, and carbohydrates in YS cells. Although in the current study we examined such mechanisms, they will not be further discussed. Nevertheless, we documented for the first time temporal gene expression patterns of all solute carrier genes (SLC genes) that were expressed in YS epithelium (Additional file 9).

### Plasma protein synthesis in the yolk sac

**Figure 6:**
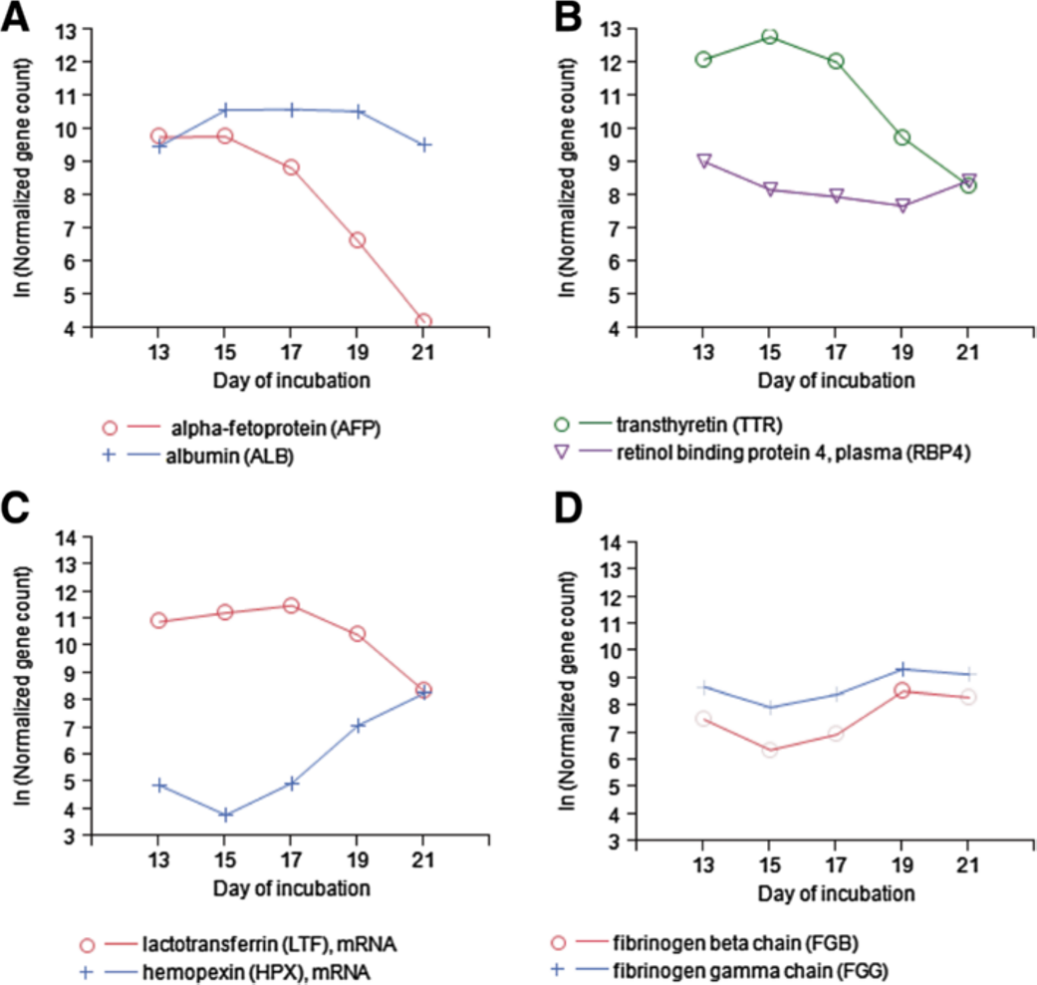
**The Yolk Sac of the chick embryo synthesizes plasma proteins normally produced by the liver.** Gene expression levels of **A**. albumin and alpha-fetoprotein, **B**. transthyretin and retinol binding protein 4, **C**. lactotransferrin and hemopexin, and **D**. selected genes related to the coagulation cascade.

The YS has been previously suggested to play a similar role as the liver in the synthesis of plasma proteins during early stages of embryonic development [36]. In the current study we found that several major plasma carrier proteins, normally produced by the liver, were among the highest expressed of all examined genes (Additional file 7). A very high expression of albumin, the major carrier protein in the blood for various cations, fatty acids, hormones, and thyroxin, was observed throughout incubation with constant levels between E15 and E19 (Figure 6A). Interestingly, the fetal protein for albumin, alfa-fetoprotein, exhibited a similar expression level as albumin on E13 and then dramatically decreased from E15 to E21 (Figure 6A), demonstrating the loss of function for fetal genes towards hatch in the YS. The blood carrier protein for thyroxin, transthyretin, exhibited very high levels of gene expression between E13 and E17 (Figure 6B), which may suggest that the YS regulates thyroxin transport to embryonic tissues. However, another possibility is that transthyretin forms a macromolecular complex in the blood together with retinol binding protein 4, which was also highly abundant in YS epithelium (Figure 6B). Both proteins are normally synthesized in the liver and their complex is essential in the mobilization of retinol (a form of vitamin A) to peripheral tissues [37].

Between E13 and E17, we observed high expression levels of lactotransferrin, an iron-binding blood plasma glycoprotein (Figure 6C). Together with the substantial up-regulation between E15 and E21 of hemopexin (Figure 6C), which binds free heme in the blood, we hypothesized that the YS may have a role in regulating blood iron levels of the chick embryo. We also found that YS epithelium expressed many of the genes coding for proteins involved in the blood coagulation cascade. Among them are fibrinogen beta and gamma chains that had a similar pattern of increase in expression between E15 and E19 (Figure 6D).

### The degradation of the yolk sac towards the end of incubation

**Figure 7:**
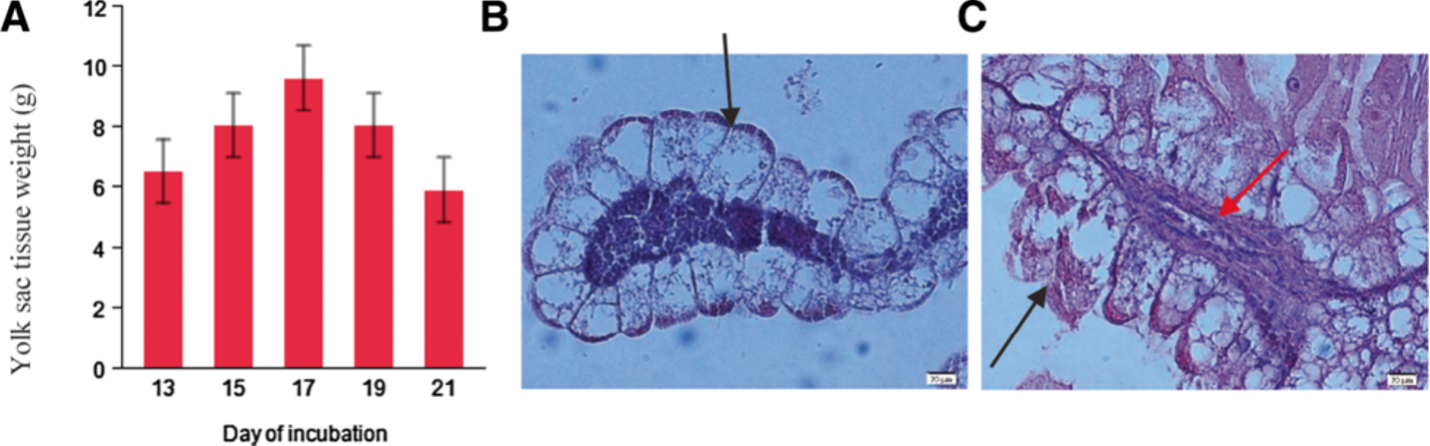
**The degradation of the Yolk Sac towards hatch. A**. Weight (g) of the YS during the last week of chick embryonic development. **B**. Histology section (H&E) of the YS on E13. **C**. Histology section (H&E) of the YS on E21. Black arrows: Endodermal epithelial cells. Red arrow: Blood vessel.

Figure 7A illustrates how the YS increases in weight between E13 and E17 and decreases in weight between E17 and E21. This is in agreement with Yadgary et al. [9] who found that YS weight and absorptive surface area (endodermal epithelial cell area) follow a pattern of increase up to E17 and decrease towards hatch. The authors speculated that the decrease is due to apoptotic processes in YS epithelium in order to enable the YS to be drawn into the body cavity of the embryo just before hatch. Here we provide histological and gene expression evidence that YS epithelial cells are degraded in the last two days of incubation. Histology sections of the YS demonstrated that the conformation of its epithelial cells changed from a distinct form of a columnar shape (Figure 7B) to an amorphous structure (Figure 7C); part of the apical membrane of these cells was degraded and their content spilled to the yolk content. Contrasts and functional annotation showed that genes associated with cytoskeleton cell structure and intermediate keratin filaments were down-regulated between E17 and E21 (Table 1). Keratin filaments in epithelial cells support the cell's structure and down regulation of several of these genes is known to be associated with susceptibility to apoptosis [38, 39]. We found that keratin 5, 14, 15 and 17 were among the top 10 down-regulated genes between E17 and E21 (Additional file 8), demonstrating the cellular pathway of degradation of the YS epithelium in the last days of incubation. It is possible that the degradation is due to YS vascular atrophy, as YS blood vessels appear to deteriorate towards hatch (Figure 7C). Further research is needed in order to elucidate the cascade of events leading to degradation of the YS epithelium. A better resolution of temporal sampling may facilitate this goal and achieve a better understanding of the processes involved in epithelial cell death.

### Hemoglobin catabolism and bile biosynthesis in YS epithelium

During the last week of chick embryonic development, the color of the residual yolk changes from yellow to green [40]. Speake and Teale [41] suggested that the change of color is due to a transfer of gallbladder bile from the embryonic intestine to the yolk through the yolk stalk, while Yadgary et al. [9] suggested that the YS produces bile components responsible for the green color of the yolk. In the current study, we observed that on E17 the residual yolk appeared green in color whereas the intestine was almost empty of contents (Additional file 10), thus supporting the latter hypothesis.

The green color of bile is primarily due to the breakdown of heme residues into biliverdin, a process that normally occurs in the liver. We speculate that a similar breakdown occurs in the epithelial cells of the YS. Evidence for that includes: 1) the increasing expression levels in YS epithelium of hemopexin, the heme-binding plasma glycoprotein (Figure 6C), 2) the up-regulation in YS epithelium of heme oxygenase, which breaks down heme into biliverdin (Additional file 10), and 3) the high and constant levels of expression for ferritin, an intracellular protein that stores iron in the liver (Additional file 10). In addition, enzymes of bile synthesis alternative pathway were expressed in the YS (Additional file 8), in agreement with Yadgary et al. [9] that detected bile acids in the yolk and YS and suggested that they are synthesized in the YS tissue. Furthermore, cysteine dioxygenase, an enzyme involved in the formation of taurine which conjugates bile acids, was the highest up-regulated gene among all examined genes in the current study- almost 700-fold between E15 and E21 (Additional file 8). Taking all that together, we suggest that red blood cells, either primitive or definitive, stemming from embryo blood circulation or from YS blood islands, are intravascularly degraded and their heme is transported to the epithelium of the YS by hemopexin. Heme is catabolized in these cells by heme oxygenase into biliverdin and iron, the iron is stored by ferritin in the YS or transported to the blood by YS derived lactotransferrin, and biliverdin is joined with conjugated bile acids that were synthesized in the YS.

It should be noted that in mammals, biliverdin is normally catabolized into bilirubin by the enzyme biliverdin reductase, however in the mature chicken bilirubin is hardly detected [42]. Here we observed that during chick embryonic development, the enzyme biliverdin reductase had moderate levels of expression on E13 which were then substantially down-regulated until hatch (Additional file 10), suggesting that in chicken embryonic development bilirubin may serve as a fetal gene with a loss of function towards hatch.

## Conclusions

Understanding YS functions and development may help achieve an optimal development of the chicken broiler embryo and a successful hatching. In the current study we completed a temporal characterization of all the genes associated with YS absorption, digestion and transport of nutrients from E13 to E21 (day of hatch). Based on our transcriptome analysis and histological sections of the YS, we conclude that:

1. Two main types of cells– erythropoietic and epithelial cells, were sampled.
2. Definitive erythropoietic cells, representing the second wave of erythropoiesis in the YS, proliferate in the YS at a very high level between E13 and E15.
3. A massive amount of YS definitive erythropoietic cells migrate to the blood circulation from E15 to E19.
4. The definitive erythropoietic cells of the YS are presumably of an embryonic origin [31, 32]. They could not have been distinguished in traditional chick/quail experiments, and therefore the YS was underestimated in its contribution to blood cell synthesis in the last week of incubation.
5. YS epithelial cells are actively involved in nutrient absorption and digestion from E13 to E21, which is carried out by digestive enzymes and transporters of fatty acids, amino acids, peptides, and carbohydrates. YS epithelial cells exhibit high levels of lipoprotein absorption, digestion and re-synthesis.
6. YS epithelial cells synthesize high levels of carrier proteins and coagulation factors from E13 to E21.
7. YS epithelial cells are actively involved in heme catabolism and bile synthesis at increasing levels from E15 to E21.
8. Towards the end of incubation, a fraction of the YS epithelial cells start degrading.

Our results support and expand current knowledge on the different functions of this unique extraembryonic tissue. The YS has a similar role as the liver in the synthesis of plasma proteins, as the bone marrow in the synthesis of new blood cells, and as the intestine in digestion of nutrients and their transport to the embryo. Thus, the YS plays different roles to support or replace the functions of several organs that have not yet reached their full functional capacity.

The current study contributes to our understanding of crucial metabolic processes during the last week of chick embryonic development as well as to early stages of mammalian embryonic development. In view of its characteristics, we suggest that the YS of the chick embryo may be used as a model to understand a wide range of processes such as fatty acid absorption and intracellular transport, nutrient transport, lysosomal digestion, apoptosis of epithelial cells, fetal gene loss of function, and blood synthesis and degradation. Furthermore, future studies that will compare high-throughput analyses between YS and different organs of the embryo, may help elucidate all of the various functional roles of the YS, which in light of the current data might be more accurately defined as an extra-embryonic organ.

## Electronic supplementary material

Additional file 1: **Number of sequenced reads per sample.** (DOCX 18 KB)

Additional file 2: **Principal component plots and outlier detection.** (DOCX 5 MB)

Additional file 3: **Functional annotation analysis to genes clustered together at several levels of partitioning.** (DOCX 5 MB)

Additional file 4: **The 50 highest expressed genes per day.** (XLSX 13 KB)

Additional file 5: **Separation and normalization of non-erythropoeitic cells' gene expression.** (DOCX 27 KB)

Additional file 6: **Separate analysis (3500 genes) - Mean and P-value of significant genes from each of the 4 contrasts.** (XLSX 216 KB)

Additional file 7: **Separate analysis (3500 genes) - The 50 highest expressed genes per day.** (XLSX 14 KB)

Additional file 8: **Separate analysis (3500 genes) - Top 50 down regulated and up regulated genes.** (XLSX 16 KB)

Additional file 9: **SLC gene expression.** (DOCX 294 KB)

Additional file 10: **YS Hemoglobin catabolism and bile biosynthesis.** (DOCX 640 KB)
