# Supplementary material for: Temporal transcriptome analysis of the chicken embryo yolk sac
Source: BMC Genomics. 2014 Aug 19;15(1):690. doi: 10.1186/1471-2164-15-690 (PMC4246430; doi:10.1186/1471-2164-15-690)
Supplement: Supplementary file 1 — Additional file 1: Number of sequenced reads per sample. (DOCX 18 KB) [file 12864_2014_6680_MOESM1_ESM.docx]

| **read count** | **sample number** | **Day of incubation** |
| --- | --- | --- |
| 7,907,780 | 1 | 13 |
| 12,044,354 | 2 | 13 |
| 7,020,980 | 3 | 13 |
| 13,545,265 | 4 | 15 |
| 9,044,889 | 5 | 15 |
| 12,308,024 | 6 | 15 |
| 17,835,384 | 7 | 17 |
| 11,625,365 | 8 | 17 |
| 12,667,144 | 9 | 17 |
| 10,789,178 | 10 | 19 |
| 18,572,771 | 11 | 19 |
| 11,993,851 | 13 | 21 |
| 18,477,035 | 14 | 21 |
| 15,168,698 | 15 | 21 |

**Number of reads per sample**
