# Supplementary figures and images for: Temporal transcriptome analysis of the chicken embryo yolk sac
Source: BMC Genomics. 2014 Aug 19;15(1):690. doi: 10.1186/1471-2164-15-690 (PMC4246430; doi:10.1186/1471-2164-15-690)

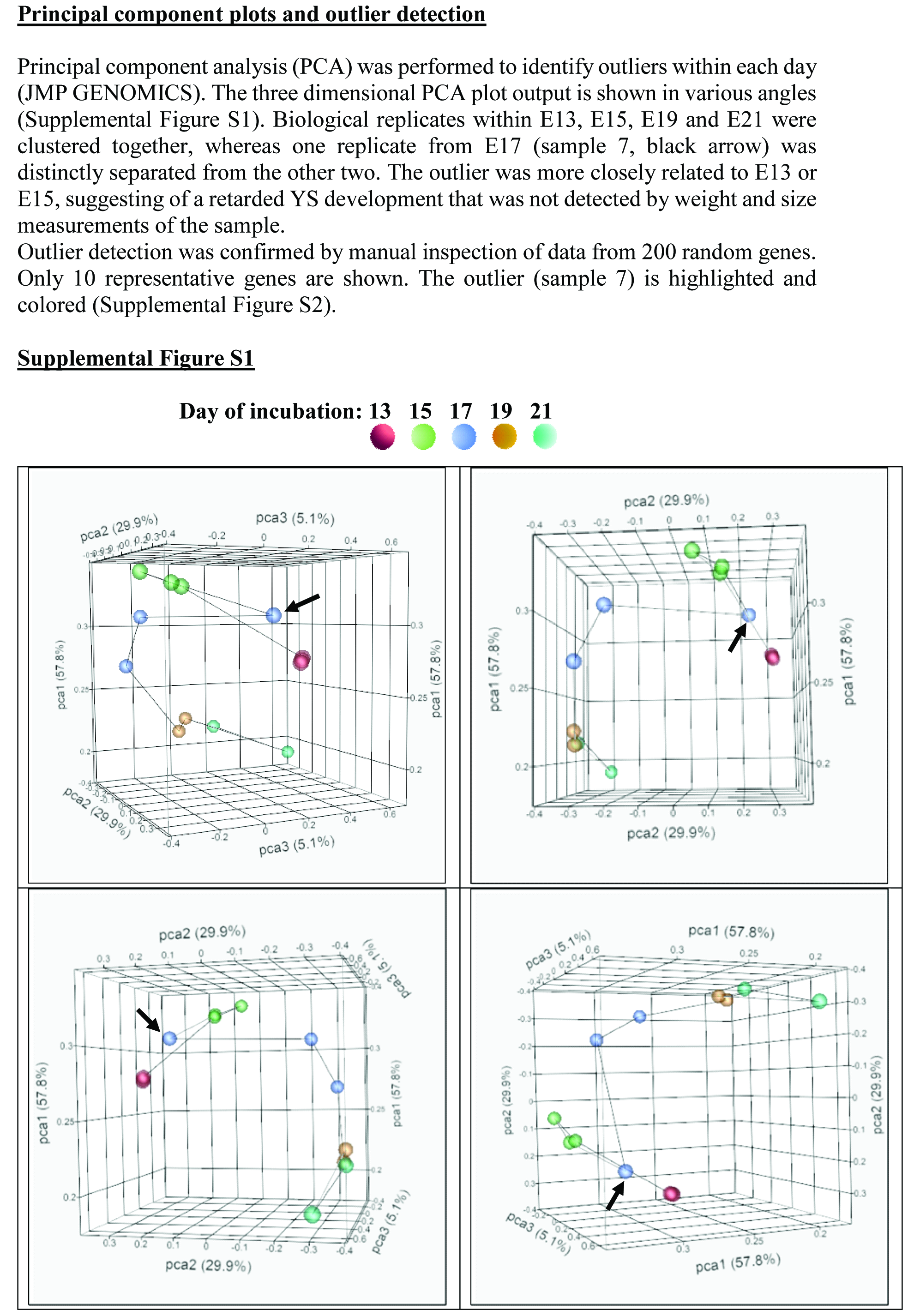

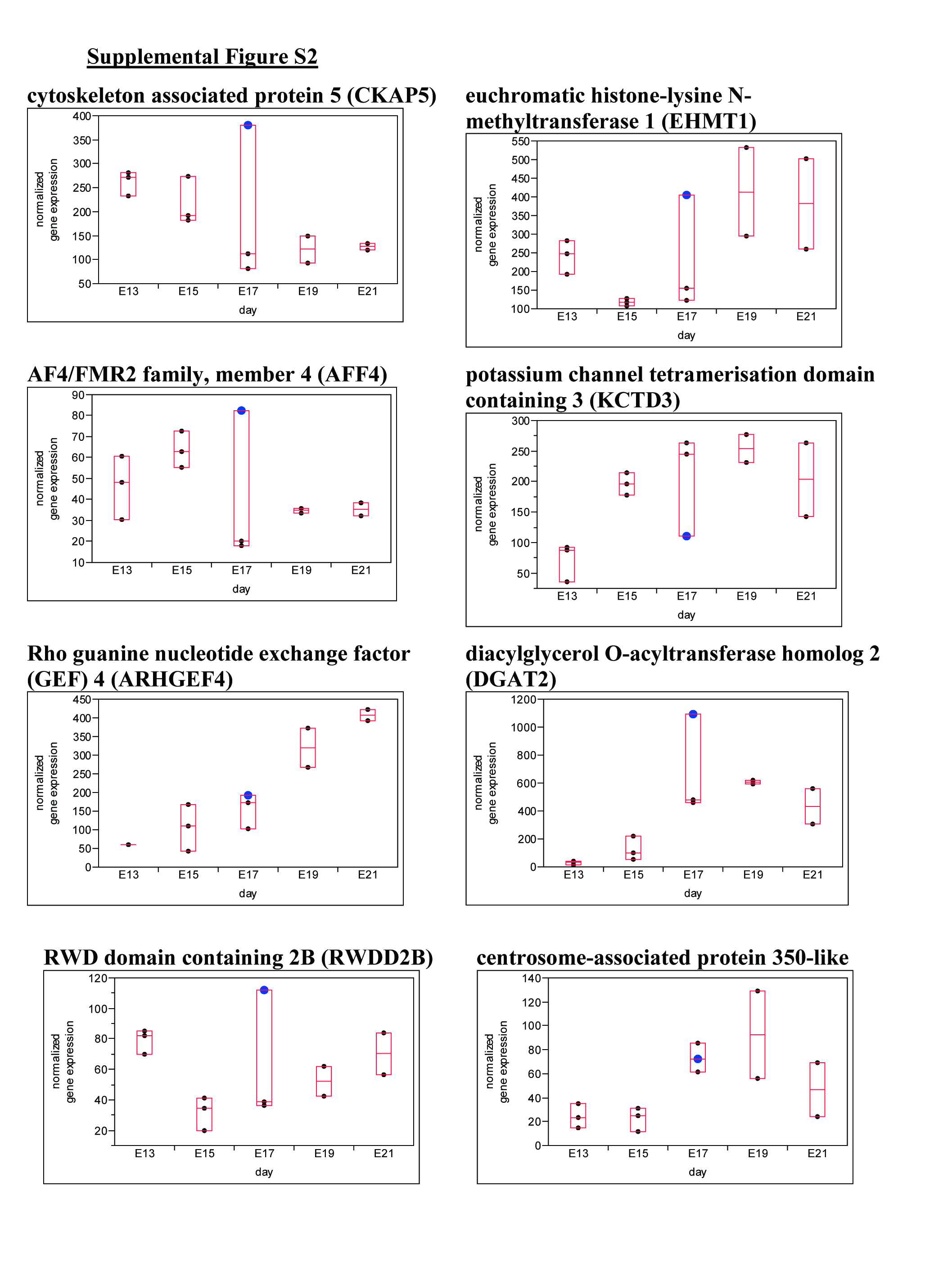

Supplement: Supplementary file 2 — Additional file 2: Principal component plots and outlier detection. (DOCX 5 MB) [file 12864_2014_6680_MOESM2_ESM.docx]

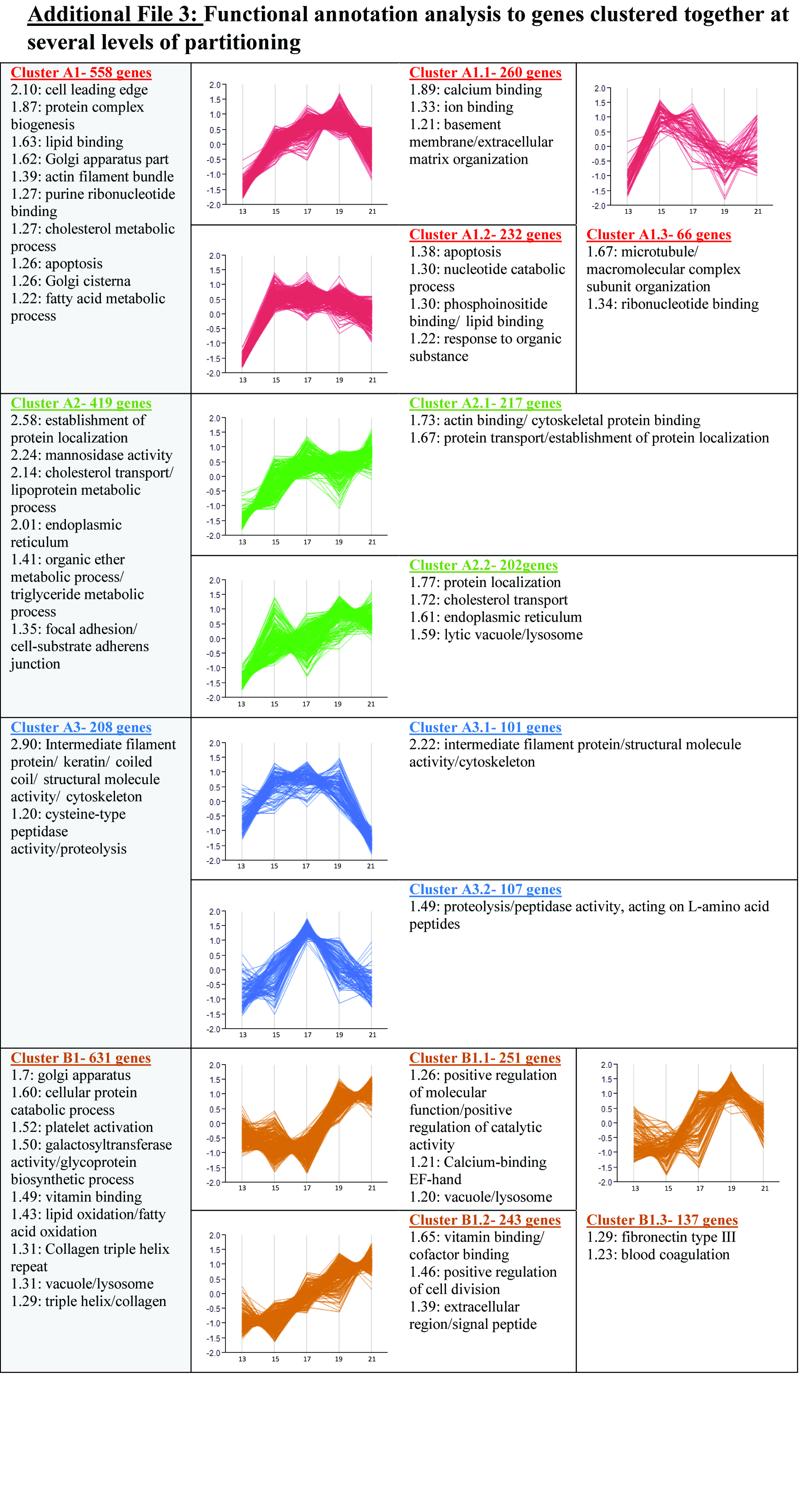

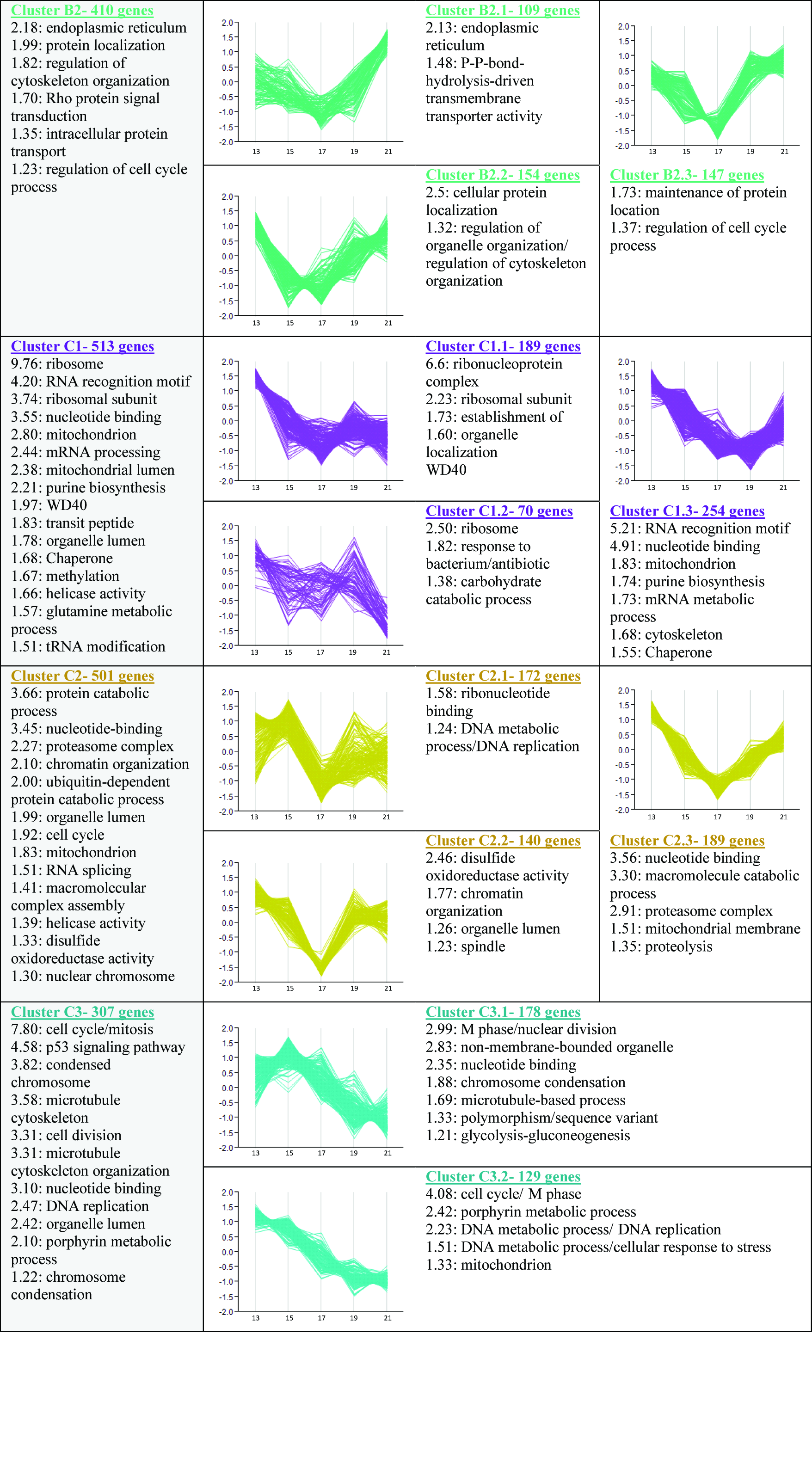

Supplement: Supplementary file 3 — Additional file 3: Functional annotation analysis to genes clustered together at several levels of partitioning. (DOCX 5 MB) [file 12864_2014_6680_MOESM3_ESM.docx]
