# Supplementary material for: Temporal transcriptome analysis of the chicken embryo yolk sac
Source: BMC Genomics. 2014 Aug 19;15(1):690. doi: 10.1186/1471-2164-15-690 (PMC4246430; doi:10.1186/1471-2164-15-690)

**Separation and normalization of non- erythropoietic cells' gene expression**

In the current study we attempted to separate the expression of at least two major cell types- the erythropoietic cells and the endodermal epithelial cells. Histology sectioning of the YS tissue revealed that the two cell types appear to have a non-even cell count during incubation with at least nine times more erythropoietic cells on E13, and after movement of these cells to the blood circulation, approximately 10 and 50 times less on E17 and E21, respectively. To retrieve genes of non-erythropoeitic cells and correct for the bias in gene expression due to uneven cell count, we first performed a new hierarchical clustering analysis on all standardized normalized 7128 genes. We used all 7128 genes and not just the significant ones because it is possible that due to lack of power and the FDR cutoff there may have been some erythropoietic genes that were not deemed as significantly down-regulated and thus were not included in the 1^st^ hierarchical clustering (Figure 2, Additional File 2).

Next, genes were chosen on the basis of their expression pattern- it is expected that cells migrating from the tissue would result in a decrease of RNA abundance for specific genes associated with these cells, such as the globin genes. Similar to the 1^st^ hierarchical clustering analysis (Figure 2, Additional File 2), there were several clusters of genes with different expression patterns. Those can be divided into three main categories depending on changes in cell count and the different levels of expression of one cell type compared to the other: clusters of genes with a strong down-regulation between E13 and E21, clusters of genes with an up-regulation or no change between E13 and E17, and clusters of genes with a strong down-regulation between E13 and E17 and then no change or an up regulation until E21. The first type of clusters represent genes of migrating erythropoetic cells that decrease in quantity. The second type of clusters represent genes of non-erythropoeitic cells, that do not increase in their number from E13 to E21 but rather increase in the number of mRNA molecules sampled and sequenced from these cells. The third type of clusters represent genes that are associated with both cell types, resulting in a sharp down-regulation of these genes as erythropoietic cells decrease in number and an up-regulation afterwards due to the increase in the number of mRNA molecules sampled from non-erythropoietic cells. It is important to mention that most genes of type 1 and type 2 clusters may also not be exclusive to a specific cell type, but rather have a much higher expression as compared to the other cell type.

We then identified genes with type 1 and type 3 expression patterns and excluded them from further analyses. The remaining 3500 genes represent mRNA expression from non-erythropoietic cells, however this expression is biased towards an up-regulation. In order to achieve a better representation of gene expression patterns for non-erythropoietic genes while adjusting for varying sequencing depth, we retrieved the raw copy number for these genes and then performed an Upper-Quartile Normalization followed by a log_e_ (ln) transformation. It is possible that not adding type 3 cluster genes affected the accuracy of gene patterns after normalization, however GO terms revealed that most genes of cluster 3 are associated with ribosome complexes and mRNA expression, and thus are expected to have a constant level of expression in epithelial cells. The effect of the normalization on the mean expression level of all 3500 selected genes during incubation is shown below:


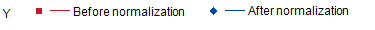


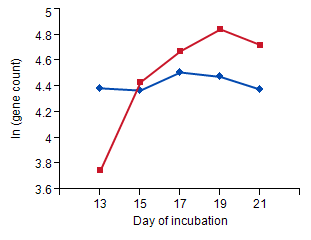

Supplement: Supplementary file 5 — Additional file 5: Separation and normalization of non-erythropoeitic cells' gene expression. (DOCX 27 KB) [file 12864_2014_6680_MOESM5_ESM.docx]
