# Supplementary material for: Temporal transcriptome analysis of the chicken embryo yolk sac
Source: BMC Genomics. 2014 Aug 19;15(1):690. doi: 10.1186/1471-2164-15-690 (PMC4246430; doi:10.1186/1471-2164-15-690)
Supplement: Supplementary file 9 — Additional file 9: SLC gene expression. (DOCX 294 KB) [file 12864_2014_6680_MOESM9_ESM.docx]

Temporal gene expression patterns of all solute carrier genes (SLC genes) that were expressed in YS epithelium (highlighted and colored). SLCs Tables are based on the SLC database (http://slc.bioparadigms.org/).

**SLC1 High-affinity glutamate and neutral amino acid transporter family**

| Gene name | Protein name | Substrates | 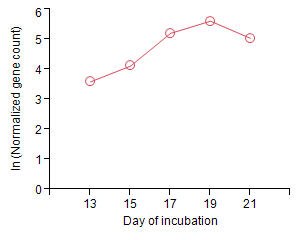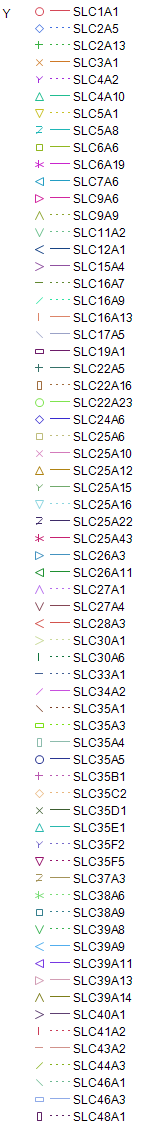 |
| --- | --- | --- | --- |
| [**SLC1A1**](http://slc.bioparadigms.org/protein?GeneName=SLC1A1) | **EAAC1, EAAT3** | **L-Glu, D/L-Asp** |  |
| [SLC1A2](http://slc.bioparadigms.org/protein?GeneName=SLC1A2) | GLT-1, EAAT2 | L-Glu, D/L-Asp |  |
| [SLC1A3](http://slc.bioparadigms.org/protein?GeneName=SLC1A3) | GLAST, EAAT1 | L-Glu, D/L-Asp |  |
| [SLC1A4](http://slc.bioparadigms.org/protein?GeneName=SLC1A4) | ASCT1, SATT | L-Ala, L-Ser, L-Cys, L-Thr |  |
| [SLC1A5](http://slc.bioparadigms.org/protein?GeneName=SLC1A5) | ASCT2, AAAT | L-Ala, L-Ser, L-Cys, L-Thr, L-Gln, L-Asn |  |
| [SLC1A6](http://slc.bioparadigms.org/protein?GeneName=SLC1A6) | EAAT4 | L-Glu, D/L-Asp |  |
| [SLC1A7](http://slc.bioparadigms.org/protein?GeneName=SLC1A7) | EAAT5 | L-Glu, D/L-Asp |  |

**SLC2 Facilitative GLUT transporter family**

| Gene name | Protein name | Substrates | 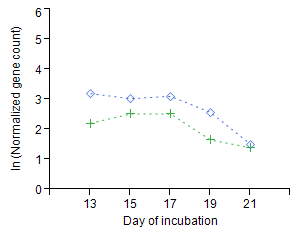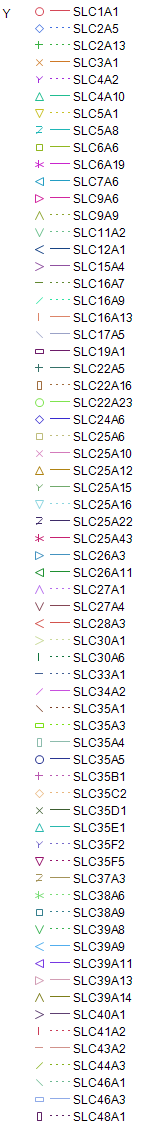 |
| --- | --- | --- | --- |
| [SLC2A1](http://slc.bioparadigms.org/protein?GeneName=SLC2A1) | GLUT1 | glucose, galactose, mannose, glucosamine |  |
| [SLC2A2](http://slc.bioparadigms.org/protein?GeneName=SLC2A2) | GLUT2 | glucose, galactose, fructose, mannose, glucosamine |  |
| [SLC2A3](http://slc.bioparadigms.org/protein?GeneName=SLC2A3) | GLUT3 | glucose, galactose, mannose, xylose |  |
| [SLC2A3P1](http://slc.bioparadigms.org/protein?GeneName=SLC2A3P1) | pseudogene |  |  |
| [SLC2A3P2](http://slc.bioparadigms.org/protein?GeneName=SLC2A3P2) | pseudogene |  |  |
| [SLC2A3P4](http://slc.bioparadigms.org/protein?GeneName=SLC2A3P4) | pseudogene |  |  |
| [SLC2A4](http://slc.bioparadigms.org/protein?GeneName=SLC2A4) | GLUT4 | glucose, glucosamine |  |
| [**SLC2A5**](http://slc.bioparadigms.org/protein?GeneName=SLC2A5) | **GLUT5** | **fructose** |  |
| [SLC2A6](http://slc.bioparadigms.org/protein?GeneName=SLC2A6) | GLUT6 | glucose |  |
| [SLC2A7](http://slc.bioparadigms.org/protein?GeneName=SLC2A7) | GLUT7 | glucose, fructose |  |
| [SLC2A8](http://slc.bioparadigms.org/protein?GeneName=SLC2A8) | GLUT8 | glucose, fructose, galactose |  |
| [SLC2A9](http://slc.bioparadigms.org/protein?GeneName=SLC2A9) | GLUT9 | urate (glucose, fructose) |  |
| [SLC2A10](http://slc.bioparadigms.org/protein?GeneName=SLC2A10) | GLUT10 | glucose, galactose |  |
| [SLC2A11](http://slc.bioparadigms.org/protein?GeneName=SLC2A11) | GLUT11 | glucose, fructose |  |
| [SLC2A12](http://slc.bioparadigms.org/protein?GeneName=SLC2A12) | GLUT12 | glucose |  |
| [**SLC2A13**](http://slc.bioparadigms.org/protein?GeneName=SLC2A13) | **HMIT** | **myo-inositol** |  |
| [SLC2A14](http://slc.bioparadigms.org/protein?GeneName=SLC2A14) | GLUT14 |  |  |
| [SLC2AXP1](http://slc.bioparadigms.org/protein?GeneName=SLC2AXP1) | pseudogene |  |  |

**SLC3 Heavy subunits of the heteromeric amino acid transporters**

| Gene name | Protein name | Substrates | 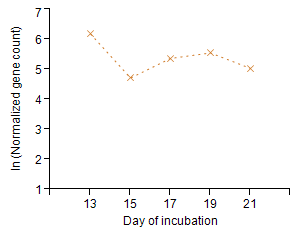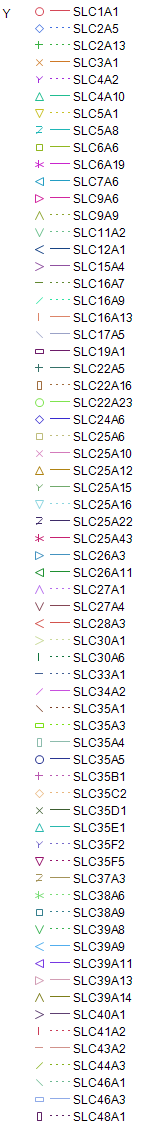 |
| --- | --- | --- | --- |
| [**SLC3A1**](http://slc.bioparadigms.org/protein?GeneName=SLC3A1) | **rBAT** | **system b0,+, heterodimerizes with light subunit SLC7A9** |  |
| [SLC3A2](http://slc.bioparadigms.org/protein?GeneName=SLC3A2) | 4F2hc | systems L, y+L, xc- and asc with light subunits SLC7A5-8 and SLC7A10-11 |  |

**SLC4 Bicarbonate transporter family**

| Gene name | Protein name | Substrates | 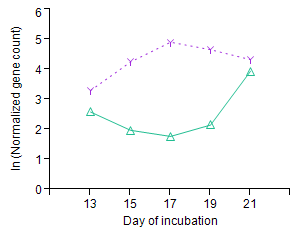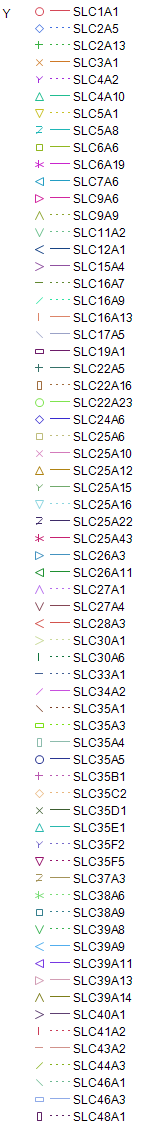 |
| --- | --- | --- | --- |
| [SLC4A1](http://slc.bioparadigms.org/protein?GeneName=SLC4A1) | AE1 | chloride cicarbonate |  |
| [**SLC4A2**](http://slc.bioparadigms.org/protein?GeneName=SLC4A2) | **AE2** | **chloride bicarbonate** |  |
| [SLC4A3](http://slc.bioparadigms.org/protein?GeneName=SLC4A3) | AE3 | chloride bicarbonate |  |
| [SLC4A4](http://slc.bioparadigms.org/protein?GeneName=SLC4A4) | NBCe1 | sodium bicarbonate (and/or carbonate) |  |
| [SLC4A5](http://slc.bioparadigms.org/protein?GeneName=SLC4A5) | NBCe2 | sodium bicarbonate (and/or carbonate) |  |
| [SLC4A7](http://slc.bioparadigms.org/protein?GeneName=SLC4A7) | NBCn1 | chloride bicarbonate |  |
| [SLC4A8](http://slc.bioparadigms.org/protein?GeneName=SLC4A8) | NDCBE | sodium bicarbonate chloride |  |
| [SLC4A9](http://slc.bioparadigms.org/protein?GeneName=SLC4A9) |  | inconclusive |  |
| [**SLC4A10**](http://slc.bioparadigms.org/protein?GeneName=SLC4A10) | **NBCn2** | **sodium bicarbonate chloride** |  |
| [SLC4A11](http://slc.bioparadigms.org/protein?GeneName=SLC4A11) | BTR1 | sodium, borate |  |

**SLC5 Sodium glucose cotransporter family**

| Gene name | Protein name | Substrates | 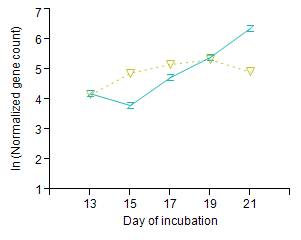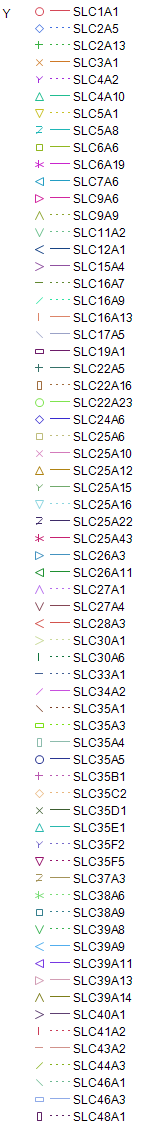 |
| --- | --- | --- | --- |
| [**SLC5A1**](http://slc.bioparadigms.org/protein?GeneName=SLC5A1) | **SGLT1** | **glucose and galactose (urea and water)** |  |
| [SLC5A2](http://slc.bioparadigms.org/protein?GeneName=SLC5A2) | SGLT2 | glucose |  |
| [SLC5A3](http://slc.bioparadigms.org/protein?GeneName=SLC5A3) | SMIT1 | myoinositol (glucose) |  |
| [SLC5A4](http://slc.bioparadigms.org/protein?GeneName=SLC5A4) | SGLT3 | Na+ (H+) |  |
| [SLC5A5](http://slc.bioparadigms.org/protein?GeneName=SLC5A5) | NIS | I- (ClO4-, SCN-, NO3-, Br-) |  |
| [SLC5A6](http://slc.bioparadigms.org/protein?GeneName=SLC5A6) | SMVT | biotin, lipoate panthothenate, I- |  |
| [SLC5A7](http://slc.bioparadigms.org/protein?GeneName=SLC5A7) | CHT | choline |  |
| [**SLC5A8**](http://slc.bioparadigms.org/protein?GeneName=SLC5A8) | **SMCT1** | **short chain fatty acids** |  |
| [SLC5A9](http://slc.bioparadigms.org/protein?GeneName=SLC5A9) | SGLT4 | mannose, fructose, glucose |  |
| [SLC5A10](http://slc.bioparadigms.org/protein?GeneName=SLC5A10) | SGLT5 | mannose, fructose, glucose |  |
| [SLC5A11](http://slc.bioparadigms.org/protein?GeneName=SLC5A11) | SMIT2 | myoinositol, chiro-inositol |  |
| [SLC5A12](http://slc.bioparadigms.org/protein?GeneName=SLC5A12) | SMCT2 | short chain fatty acids |  |

**SLC6 Sodium- and chloride-dependent neurotransmitter transporter family**

| Gene name | Protein name | Substrates | 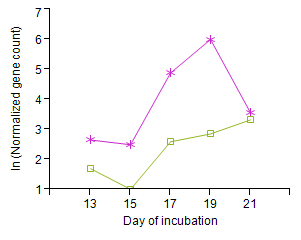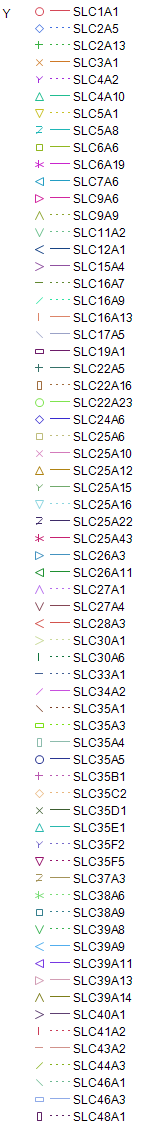 |
| --- | --- | --- | --- |
| [SLC6A1](http://slc.bioparadigms.org/protein?GeneName=SLC6A1) |  | GABA |  |
| [SLC6A2](http://slc.bioparadigms.org/protein?GeneName=SLC6A2) |  | norepinephrine |  |
| [SLC6A3](http://slc.bioparadigms.org/protein?GeneName=SLC6A3) |  | dopamine |  |
| [SLC6A4](http://slc.bioparadigms.org/protein?GeneName=SLC6A4) |  | serotonin |  |
| [SLC6A5](http://slc.bioparadigms.org/protein?GeneName=SLC6A5) |  | glycine |  |
| [**SLC6A6**](http://slc.bioparadigms.org/protein?GeneName=SLC6A6) |  | **taurine** |  |
| [SLC6A7](http://slc.bioparadigms.org/protein?GeneName=SLC6A7) |  | proline |  |
| [SLC6A8](http://slc.bioparadigms.org/protein?GeneName=SLC6A8) |  | creatine |  |
| [SLC6A9](http://slc.bioparadigms.org/protein?GeneName=SLC6A9) |  | glycine |  |
| [SLC6A10](http://slc.bioparadigms.org/protein?GeneName=SLC6A10) |  |  |  |
| [SLC6A10P](http://slc.bioparadigms.org/protein?GeneName=SLC6A10P) | pseudogene |  |  |
| [SLC6A11](http://slc.bioparadigms.org/protein?GeneName=SLC6A11) |  | GABA |  |
| [SLC6A12](http://slc.bioparadigms.org/protein?GeneName=SLC6A12) |  | betaine, GABA |  |
| [SLC6A13](http://slc.bioparadigms.org/protein?GeneName=SLC6A13) |  | GABA |  |
| [SLC6A14](http://slc.bioparadigms.org/protein?GeneName=SLC6A14) |  | neutral, cationic amino acids |  |
| [SLC6A15](http://slc.bioparadigms.org/protein?GeneName=SLC6A15) |  | large, neutral amino acids |  |
| [SLC6A16](http://slc.bioparadigms.org/protein?GeneName=SLC6A16) |  | unknown |  |
| [SLC6A17](http://slc.bioparadigms.org/protein?GeneName=SLC6A17) |  | neutral amino acids |  |
| [SLC6A18](http://slc.bioparadigms.org/protein?GeneName=SLC6A18) |  | neutral amino acids |  |
| [**SLC6A19**](http://slc.bioparadigms.org/protein?GeneName=SLC6A19) |  | **neutral amino acids** |  |
| [SLC6A20](http://slc.bioparadigms.org/protein?GeneName=SLC6A20) |  | proline, pipecolate, sarcosine |  |
| [SLC6A21](http://slc.bioparadigms.org/protein?GeneName=SLC6A21) | pseudogene |  |  |

**SLC7 Cationic amino acid transporter/glycoprotein-associated family**

| Gene name | Protein name | Substrates | 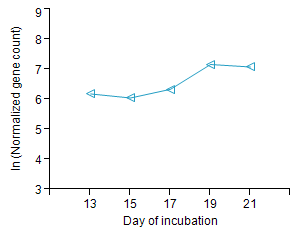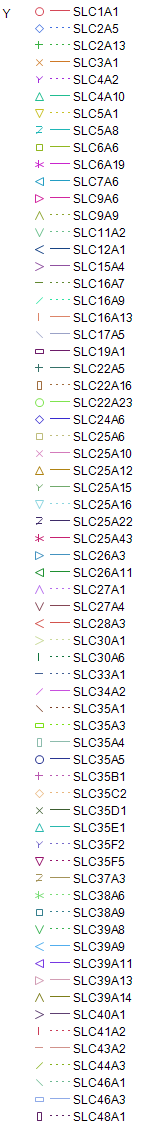 |
| --- | --- | --- | --- |
| [SLC7A1](http://slc.bioparadigms.org/protein?GeneName=SLC7A1) | CAT-1 | cationic L-amino acids |  |
| [SLC7A2](http://slc.bioparadigms.org/protein?GeneName=SLC7A2) | CAT-2 (A or B) | cationic L-amino acids |  |
| [SLC7A3](http://slc.bioparadigms.org/protein?GeneName=SLC7A3) | CAT-3 | cationic L-amino acids |  |
| [SLC7A4](http://slc.bioparadigms.org/protein?GeneName=SLC7A4) | CAT-4 |  |  |
| [SLC7A5](http://slc.bioparadigms.org/protein?GeneName=SLC7A5) | LAT1 | large neutral L-amino acids, T3, T4, L-DOPA, BCH |  |
| [SLC7A5P1](http://slc.bioparadigms.org/protein?GeneName=SLC7A5P1) | pseudogene |  |  |
| [**SLC7A6**](http://slc.bioparadigms.org/protein?GeneName=SLC7A6) | **y+LAT2** | **cationic amino acids (Na+ indep.), large neutral L-amino acids (Na+ dep.)** |  |
| [SLC7A7](http://slc.bioparadigms.org/protein?GeneName=SLC7A7) | y+LAT1 | cationic amino acids (Na+ indep.), large neutral L-amino acids (Na+ dep.) |  |
| [SLC7A8](http://slc.bioparadigms.org/protein?GeneName=SLC7A8) | LAT2 | neutral L-amino acids, T3, T4, BCH |  |
| [SLC7A9](http://slc.bioparadigms.org/protein?GeneName=SLC7A9) | b0,+AT | cationic amino acids, large neutral amino acids |  |
| [SLC7A10](http://slc.bioparadigms.org/protein?GeneName=SLC7A10) | Asc-1 | small neutral amino acids |  |
| [SLC7A11](http://slc.bioparadigms.org/protein?GeneName=SLC7A11) | xCT | cystine (anionic form), L-glutamate |  |
| [SLC7A13](http://slc.bioparadigms.org/protein?GeneName=SLC7A13) | AGT-1 | L-aspartate, L-glutamate |  |
| [SLC7A14](http://slc.bioparadigms.org/protein?GeneName=SLC7A14) |  |  |  |
| [SLC7A15P](http://slc.bioparadigms.org/protein?GeneName=SLC7A15P) | pseudogene |  |  |

**SLC8 Na+/Ca2+ exchanger family**

| Gene name | Protein name | Substrates |
| --- | --- | --- |
| [SLC8A1](http://slc.bioparadigms.org/protein?GeneName=SLC8A1) | NCX1 | Na+, Ca2+ |
| [SLC8A2](http://slc.bioparadigms.org/protein?GeneName=SLC8A2) | NCX2 | Na+, Ca2+ |
| [SLC8A3](http://slc.bioparadigms.org/protein?GeneName=SLC8A3) | NCX3 | Na+, Ca2+ |
| [SLC8B1](http://slc.bioparadigms.org/protein?GeneName=SLC8B1) | NCLX | Na+, Li+, Ca2+ |

**SLC9 Na+/H+ exchanger family**

| Gene name | Protein name | Substrates | 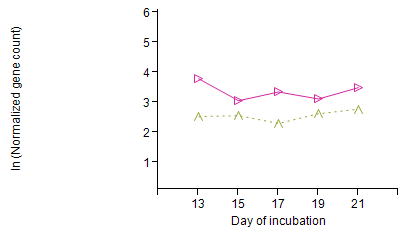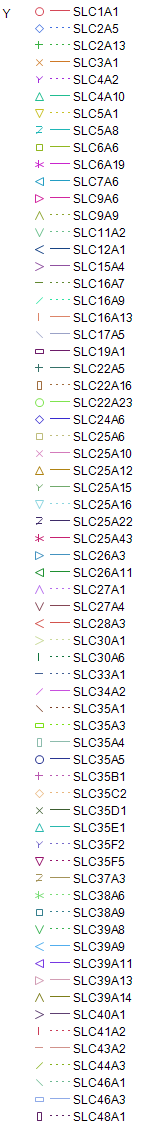 |
| --- | --- | --- | --- |
| [SLC9A1](http://slc.bioparadigms.org/protein?GeneName=SLC9A1) | NHE1 | Na+, Li+, H+, NH4+ |  |
| [SLC9A2](http://slc.bioparadigms.org/protein?GeneName=SLC9A2) | NHE2 | Na+, Li+, H+, NH4+ |  |
| [SLC9A3](http://slc.bioparadigms.org/protein?GeneName=SLC9A3) | NHE3 | Na+, Li+, H+, NH4+ |  |
| [SLC9A3P1](http://slc.bioparadigms.org/protein?GeneName=SLC9A3P1) | pseudogene |  |  |
| [SLC9A3P2](http://slc.bioparadigms.org/protein?GeneName=SLC9A3P2) | pseudogene |  |  |
| [SLC9A3P3](http://slc.bioparadigms.org/protein?GeneName=SLC9A3P3) | pseudogene |  |  |
| [SLC9A3P4](http://slc.bioparadigms.org/protein?GeneName=SLC9A3P4) | pseudogene |  |  |
| [SLC9A4](http://slc.bioparadigms.org/protein?GeneName=SLC9A4) | NHE4 | Na+, Li+ (?), H+, NH4+ |  |
| [SLC9A5](http://slc.bioparadigms.org/protein?GeneName=SLC9A5) | NHE5 | Na+, Li+, H+, NH4+ (?) |  |
| [**SLC9A6**](http://slc.bioparadigms.org/protein?GeneName=SLC9A6) | **NHE6** | **Na+, K+, H+** |  |
| [SLC9A7](http://slc.bioparadigms.org/protein?GeneName=SLC9A7) | NHE7 | Na+, K+, Li+, H+, NH4+ (?) |  |
| [SLC9A7P1](http://slc.bioparadigms.org/protein?GeneName=SLC9A7P1) | pseudogene, partially processed |  |  |
| [SLC9A8](http://slc.bioparadigms.org/protein?GeneName=SLC9A8) | NHE8 | Na+, K+, H+ |  |
| [**SLC9A9**](http://slc.bioparadigms.org/protein?GeneName=SLC9A9) | **NHE9** | **Na+, K+, H+** |  |
| [SLC9B1](http://slc.bioparadigms.org/protein?GeneName=SLC9B1) | NHA1 (NHEDC1) |  |  |
| [SLC9B2](http://slc.bioparadigms.org/protein?GeneName=SLC9B2) | NHA2 (NHEDC2) | Na+, Li+ |  |
| [SLC9C1](http://slc.bioparadigms.org/protein?GeneName=SLC9C1) | Sperm-NHE | Na+, H+ |  |
| [SLC9C2](http://slc.bioparadigms.org/protein?GeneName=SLC9C2) |  |  |  |

**SLC10 Sodium bile salt cotransport family**

| Gene name | Protein name | Substrates |
| --- | --- | --- |
| [SLC10A1](http://slc.bioparadigms.org/protein?GeneName=SLC10A1) | NTCP | bile acids |
| [SLC10A2](http://slc.bioparadigms.org/protein?GeneName=SLC10A2) | ASBT | bile acids |
| [SLC10A3](http://slc.bioparadigms.org/protein?GeneName=SLC10A3) | P3 |  |
| [SLC10A4](http://slc.bioparadigms.org/protein?GeneName=SLC10A4) | P4 |  |
| [SLC10A5](http://slc.bioparadigms.org/protein?GeneName=SLC10A5) | P5 |  |
| [SLC10A6](http://slc.bioparadigms.org/protein?GeneName=SLC10A6) | SOAT | estrone-3-sulfate, dehydroepiandrosterone sulfate, pregnenolone sulfate |
| [SLC10A7](http://slc.bioparadigms.org/protein?GeneName=SLC10A7) | P7 |  |

**SLC11 Proton-coupled metal ion transporter family**

| Gene name | Protein name | Substrates | 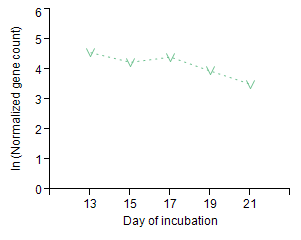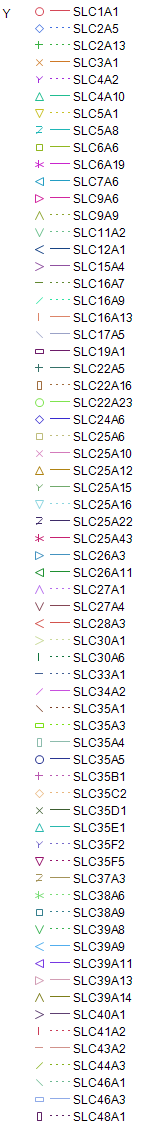 |
| --- | --- | --- | --- |
| [SLC11A1](http://slc.bioparadigms.org/protein?GeneName=SLC11A1) | NRAMP1 | Mn2+, Fe2+, other divalent metal ions |  |
| [**SLC11A2**](http://slc.bioparadigms.org/protein?GeneName=SLC11A2) | **DMT1** | **Fe2+, Cd2+, Co2+, Cu1+, Mn2+, Ni2+, Pb2+, Zn2+** |  |

**SLC12 Electroneutral cation-coupled Cl cotransporter family**

| Gene name | Protein name | Substrates | 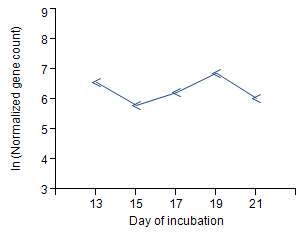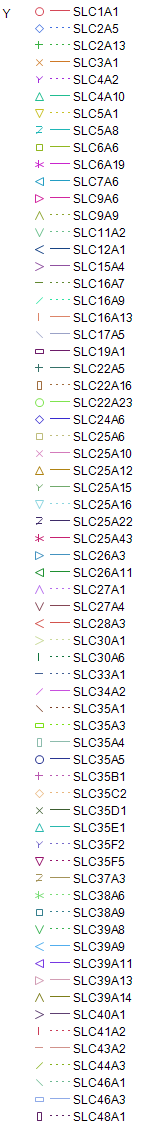 |
| --- | --- | --- | --- |
| [**SLC12A1**](http://slc.bioparadigms.org/protein?GeneName=SLC12A1) | **NKCC2** | **Na+, K+, Cl-** |  |
| [SLC12A2](http://slc.bioparadigms.org/protein?GeneName=SLC12A2) | NKCC1 | Na+, K+, Cl- |  |
| [SLC12A3](http://slc.bioparadigms.org/protein?GeneName=SLC12A3) | NCC | Na+, Cl- |  |
| [SLC12A4](http://slc.bioparadigms.org/protein?GeneName=SLC12A4) | KCC1 | K+, Cl- |  |
| [SLC12A5](http://slc.bioparadigms.org/protein?GeneName=SLC12A5) | KCC2 | K+, Cl- |  |
| [SLC12A6](http://slc.bioparadigms.org/protein?GeneName=SLC12A6) | KCC3 | K+, Cl- |  |
| [SLC12A7](http://slc.bioparadigms.org/protein?GeneName=SLC12A7) | KCC4 | K+, Cl- |  |
| [SLC12A8](http://slc.bioparadigms.org/protein?GeneName=SLC12A8) | CCC9 | unknown |  |
| [SLC12A9](http://slc.bioparadigms.org/protein?GeneName=SLC12A9) | CIP | polyamines? |  |

**SLC13 Human Na+-sulfate/carboxylate cotransporter family**

| Gene name | Protein name | Substrates |
| --- | --- | --- |
| [SLC13A1](http://slc.bioparadigms.org/protein?GeneName=SLC13A1) | NaS1 | sulfate, selenate, thiosulfate |
| [SLC13A2](http://slc.bioparadigms.org/protein?GeneName=SLC13A2) | NaC1 | succinate, citrate, ?-ketoglutarate |
| [SLC13A3](http://slc.bioparadigms.org/protein?GeneName=SLC13A3) | NaC3 | succinate, citrate, ?-ketoglutarate, NALA, glutarate and its derivatives |
| [SLC13A4](http://slc.bioparadigms.org/protein?GeneName=SLC13A4) | NaS2 | sulfate, oxyanions selenium and chromium |
| [SLC13A5](http://slc.bioparadigms.org/protein?GeneName=SLC13A5) | NaC2 | citrate, succinate, pyruvate |

**SLC14 Urea transporter family**

| Gene name |
| --- |
| [SLC14A1_UT-B1](http://slc.bioparadigms.org/protein?GeneName=SLC14A1_UT-B1) |
| [SLC14A1_UT-B2](http://slc.bioparadigms.org/protein?GeneName=SLC14A1_UT-B2) |
| [SLC14A2_UT-A1](http://slc.bioparadigms.org/protein?GeneName=SLC14A2_UT-A1) |
| [SLC14A2_UT-A2](http://slc.bioparadigms.org/protein?GeneName=SLC14A2_UT-A2) |
| [SLC14A2_UT-A3](http://slc.bioparadigms.org/protein?GeneName=SLC14A2_UT-A3) |
| [SLC14A2_UT-A4](http://slc.bioparadigms.org/protein?GeneName=SLC14A2_UT-A4) |
| [SLC14A2_UT-A5](http://slc.bioparadigms.org/protein?GeneName=SLC14A2_UT-A5) |
| [SLC14A2_UT-A6](http://slc.bioparadigms.org/protein?GeneName=SLC14A2_UT-A6) |

**SLC15 Proton oligopeptide cotransporter family**

| Gene name | Protein name | Substrates | 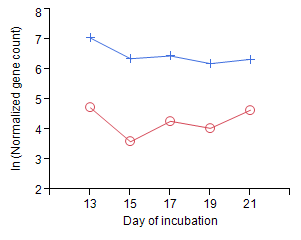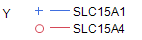 |
| --- | --- | --- | --- |
| [**SLC15A1**](http://slc.bioparadigms.org/protein?GeneName=SLC15A1) | **PEPT1** | **di- and tri-peptides, protons, beta-lactam antibiotics** |  |
| [SLC15A2](http://slc.bioparadigms.org/protein?GeneName=SLC15A2) | PEPT2 | di- and tri-peptides, protons, beta-lactam antibiotics |  |
| [SLC15A3](http://slc.bioparadigms.org/protein?GeneName=SLC15A3) | PHT2 | di- and tri-peptides, protons, histidine |  |
| [**SLC15A4**](http://slc.bioparadigms.org/protein?GeneName=SLC15A4) | **PHT1** | **di- and tri-peptides, protons, histidine** |  |

**SLC16 Monocarboxylate transporter family**

| Gene name | Protein name | Substrates | 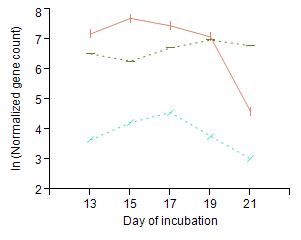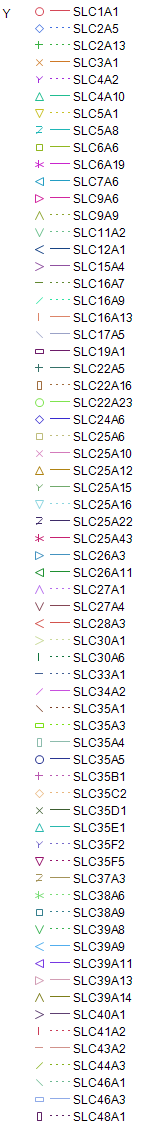 |
| --- | --- | --- | --- |
| [SLC16A1](http://slc.bioparadigms.org/protein?GeneName=SLC16A1) | MCT1, | lactate, pyruvate, ketone bodies |  |
| [SLC16A2](http://slc.bioparadigms.org/protein?GeneName=SLC16A2) | MCT8 | T2, rT3, T3, T4 |  |
| [SLC16A3](http://slc.bioparadigms.org/protein?GeneName=SLC16A3) | MCT4 | lactate, ketone bodies |  |
| [SLC16A4](http://slc.bioparadigms.org/protein?GeneName=SLC16A4) | MCT5 |  |  |
| [SLC16A5](http://slc.bioparadigms.org/protein?GeneName=SLC16A5) | MCT6 | bumetanide, probenecid, nateglinide ? |  |
| [SLC16A6](http://slc.bioparadigms.org/protein?GeneName=SLC16A6) | MCT7 |  |  |
| [**SLC16A7**](http://slc.bioparadigms.org/protein?GeneName=SLC16A7) | **MCT2** | **pyruvate, lactate, ketone bodies** |  |
| [SLC16A8](http://slc.bioparadigms.org/protein?GeneName=SLC16A8) | MCT3 | lactate |  |
| [**SLC16A9**](http://slc.bioparadigms.org/protein?GeneName=SLC16A9) | **MCT9** |  |  |
| [SLC16A10](http://slc.bioparadigms.org/protein?GeneName=SLC16A10) | TAT1, MCT10 | aromatic amino acids, T3, T4 |  |
| [SLC16A11](http://slc.bioparadigms.org/protein?GeneName=SLC16A11) | MCT11 |  |  |
| [SLC16A12](http://slc.bioparadigms.org/protein?GeneName=SLC16A12) | MCT12 |  |  |
| [SLC16A13](http://slc.bioparadigms.org/protein?GeneName=SLC16A13) | MCT13 |  |  |
| [**SLC16A14**](http://slc.bioparadigms.org/protein?GeneName=SLC16A14) | **MCT14** |  |  |

**SLC17 Vesicular glutamate transporter family**

| Gene name | Protein name | Substrates | 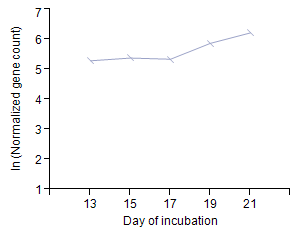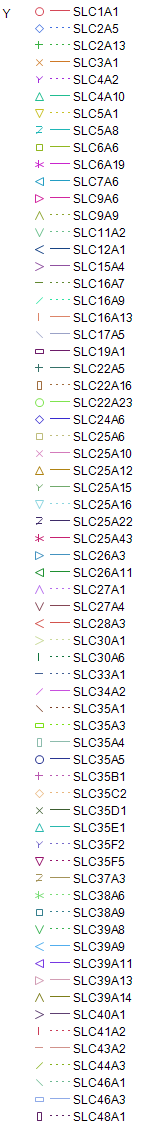 |
| --- | --- | --- | --- |
| [SLC17A1](http://slc.bioparadigms.org/protein?GeneName=SLC17A1) | NPT1 | organic anions, phosphate, chloride |  |
| [SLC17A2](http://slc.bioparadigms.org/protein?GeneName=SLC17A2) | NPT3 | unknown |  |
| [SLC17A3](http://slc.bioparadigms.org/protein?GeneName=SLC17A3) | NPT4 | organic anions |  |
| [SLC17A4](http://slc.bioparadigms.org/protein?GeneName=SLC17A4) | Na+/PO4-cotransporter homologue | unknown |  |
| [**SLC17A5**](http://slc.bioparadigms.org/protein?GeneName=SLC17A5) | **sialin** | **sialic acid, other acidic sugars** |  |
| [SLC17A6](http://slc.bioparadigms.org/protein?GeneName=SLC17A6) | VGLUT2 | glutamate |  |
| [SLC17A7](http://slc.bioparadigms.org/protein?GeneName=SLC17A7) | VGLUT1 | glutamate |  |
| [SLC17A8](http://slc.bioparadigms.org/protein?GeneName=SLC17A8) | VGLUT3 | glutamate |  |
| [SLC17A9](http://slc.bioparadigms.org/protein?GeneName=SLC17A9) | VNUT | purine nucleotides |  |

**SLC18 Vesicular amine transporter family**

| Gene name | Protein name | Substrates |
| --- | --- | --- |
| [SLC18A1](http://slc.bioparadigms.org/protein?GeneName=SLC18A1) | VMAT1 | 5-HT, DA, NE, epinephrine |
| [SLC18A2](http://slc.bioparadigms.org/protein?GeneName=SLC18A2) | VMAT2 | 5-HT, DA, NE, epinephrine, histamine |
| [SLC18A3](http://slc.bioparadigms.org/protein?GeneName=SLC18A3) | VAChT | acetylcholine |
| [SLC18B1](http://slc.bioparadigms.org/protein?GeneName=SLC18B1) | C6orf192 | unknown |

**SLC19 Folate/thiamine transporter family**

| Gene name | Protein name | Substrates | 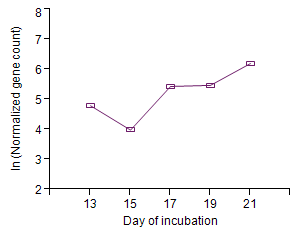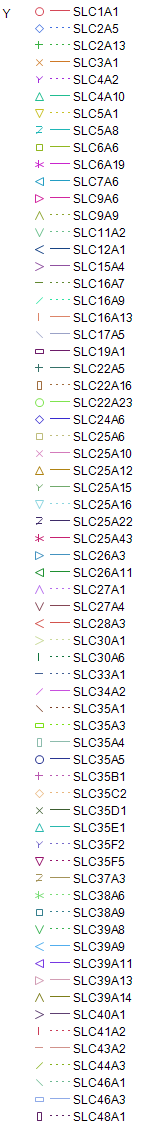 |
| --- | --- | --- | --- |
| [**SLC19A1**](http://slc.bioparadigms.org/protein?GeneName=SLC19A1) | **RFC** | **reduced folates, antifolates** |  |
| [SLC19A2](http://slc.bioparadigms.org/protein?GeneName=SLC19A2) | THTR1 | thiamine |  |
| [SLC19A3](http://slc.bioparadigms.org/protein?GeneName=SLC19A3) | THTR2 | thiamine |  |

**SLC20 Type III Na+-phosphate cotransporter family**

| Gene name | Protein name | Substrates |
| --- | --- | --- |
| [SLC20A1](http://slc.bioparadigms.org/protein?GeneName=SLC20A1) | PiT-1 | inorganic phosphate (monovalent) |
| [SLC20A2](http://slc.bioparadigms.org/protein?GeneName=SLC20A2) | PiT-2 | inorganic phosphate (monovalent) |

**SLC21 Organic anion transporter family**

| Gene name | Protein name | Substrates |
| --- | --- | --- |
| [SLCO1A2](http://slc.bioparadigms.org/protein?GeneName=SLCO1A2) | OATP1A2 | bile salts, organic anions and cations |
| [SLCO1B1](http://slc.bioparadigms.org/protein?GeneName=SLCO1B1) | OATP1B1 | bile salts, organic anions |
| [SLCO1B3](http://slc.bioparadigms.org/protein?GeneName=SLCO1B3) | OATP1B3 | bile salts, organic anions |
| [SLCO1C1](http://slc.bioparadigms.org/protein?GeneName=SLCO1C1) | OATP1C1 | T4, T3, rT3 |
| [SLCO2A1](http://slc.bioparadigms.org/protein?GeneName=SLCO2A1) | OATP2A1 | prostaglandins (C/lactate) |
| [SLCO2B1](http://slc.bioparadigms.org/protein?GeneName=SLCO2B1) | OATP2B1 | E-3-S, DHEAS |
| [SLCO3A1](http://slc.bioparadigms.org/protein?GeneName=SLCO3A1) |  |  |
| [SLCO4A1](http://slc.bioparadigms.org/protein?GeneName=SLCO4A1) |  |  |
| [SLCO4C1](http://slc.bioparadigms.org/protein?GeneName=SLCO4C1) |  |  |
| [SLCO5A1](http://slc.bioparadigms.org/protein?GeneName=SLCO5A1) |  |  |
| [SLCO6A1](http://slc.bioparadigms.org/protein?GeneName=SLCO6A1) |  |  |

**SLC22 Organic cation/anion/zwitterion transporter family**

| Gene name | Protein name | Substrates | 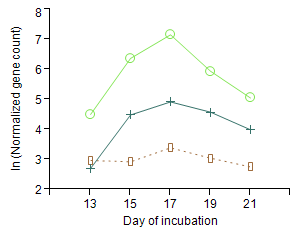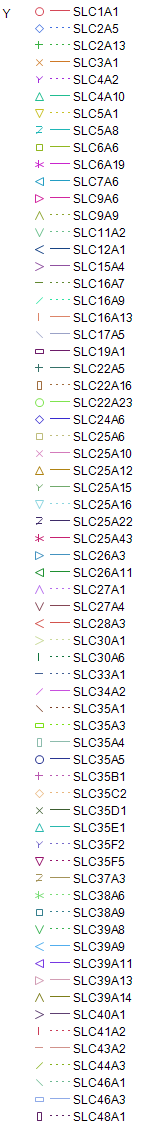 |
| --- | --- | --- | --- |
| [SLC22A1](http://slc.bioparadigms.org/protein?GeneName=SLC22A1) | OCT1 | organic cations |  |
| [SLC22A2](http://slc.bioparadigms.org/protein?GeneName=SLC22A2) | OCT2 | organic cations |  |
| [SLC22A3](http://slc.bioparadigms.org/protein?GeneName=SLC22A3) | OCT2 | organic cations |  |
| [SLC22A4](http://slc.bioparadigms.org/protein?GeneName=SLC22A4) | OCTN1 | zwitterions, organic cations |  |
| [**SLC22A5**](http://slc.bioparadigms.org/protein?GeneName=SLC22A5) | **OCTN2** | **zwitterions (L-carnitine), organic cations** |  |
| [SLC22A6](http://slc.bioparadigms.org/protein?GeneName=SLC22A6) | OAT1 | organic anions |  |
| [SLC22A7](http://slc.bioparadigms.org/protein?GeneName=SLC22A7) | OAT2 | organic anions |  |
| [SLC22A8](http://slc.bioparadigms.org/protein?GeneName=SLC22A8) | OAT3 | organic anions |  |
| [SLC22A9](http://slc.bioparadigms.org/protein?GeneName=SLC22A9) | OAT7 | organic anions |  |
| [SLC22A10](http://slc.bioparadigms.org/protein?GeneName=SLC22A10) | OAT5 | not determined |  |
| [SLC22A11](http://slc.bioparadigms.org/protein?GeneName=SLC22A11) | OAT4 | organic anions |  |
| [SLC22A12](http://slc.bioparadigms.org/protein?GeneName=SLC22A12) | URAT1 | urate, organic anions |  |
| [SLC22A13](http://slc.bioparadigms.org/protein?GeneName=SLC22A13) | OAT10 | urate, organic anions |  |
| [SLC22A14](http://slc.bioparadigms.org/protein?GeneName=SLC22A14) | OCTL2 | not determined |  |
| [SLC22A15](http://slc.bioparadigms.org/protein?GeneName=SLC22A15) | FLIPT1 | not determined |  |
| [**SLC22A16**](http://slc.bioparadigms.org/protein?GeneName=SLC22A16) | **39726** | **L-carnitine, noncharged compounds** |  |
| [SLC22A17](http://slc.bioparadigms.org/protein?GeneName=SLC22A17) | BOIT | not determined |  |
| [SLC22A18](http://slc.bioparadigms.org/protein?GeneName=SLC22A18) |  | probably organic anions |  |
| [SLC22A20](http://slc.bioparadigms.org/protein?GeneName=SLC22A20) | OAT6 | probably organic anions |  |
| [**SLC22A23**](http://slc.bioparadigms.org/protein?GeneName=SLC22A23) |  |  |  |
| [SLC22A24](http://slc.bioparadigms.org/protein?GeneName=SLC22A24) |  |  |  |
| [SLC22A25](http://slc.bioparadigms.org/protein?GeneName=SLC22A25) | UST6 |  |  |
| [SLC22A31](http://slc.bioparadigms.org/protein?GeneName=SLC22A31) |  |  |  |

**SLC23 Na+-dependent ascorbic acid transporter family**

| Gene name | Protein name | Substrates |
| --- | --- | --- |
| [SLC23A1](http://slc.bioparadigms.org/protein?GeneName=SLC23A1) | SVCT1 | L-ascorbic acid |
| [SLC23A2](http://slc.bioparadigms.org/protein?GeneName=SLC23A2) | SVCT2 | L-ascorbic acid |
| [SLC23A3](http://slc.bioparadigms.org/protein?GeneName=SLC23A3) | SVCT3 |  |
| [SLC23A4P](http://slc.bioparadigms.org/protein?GeneName=SLC23A4P) | SVCT4 |  |

**SLC24 Na+/(Ca2+-K+) exchanger family**

| Gene name | Protein name | Substrates | 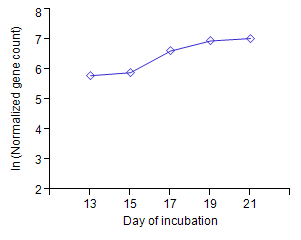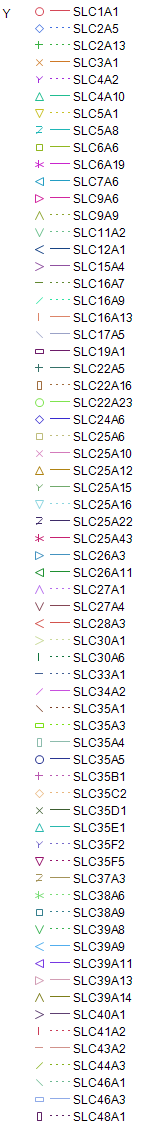 |
| --- | --- | --- | --- |
| [SLC24A1](http://slc.bioparadigms.org/protein?GeneName=SLC24A1) | NCKX1 | Na+, Ca2+, K+ |  |
| [SLC24A2](http://slc.bioparadigms.org/protein?GeneName=SLC24A2) | NCKX2 | Na+, Ca2+, K+ |  |
| [SLC24A3](http://slc.bioparadigms.org/protein?GeneName=SLC24A3) | NCKX3 | Na+, Ca2+, K+ |  |
| [SLC24A4](http://slc.bioparadigms.org/protein?GeneName=SLC24A4) | NCKX4 | Na+, Ca2+, K+ |  |
| [SLC24A5](http://slc.bioparadigms.org/protein?GeneName=SLC24A5) | NCKX5 | Na+, Ca2+, K+ |  |
| [**SLC24A**](http://slc.bioparadigms.org/protein?GeneName=SLC24A5)**6** |  |  |  |

**SLC25 Mitochondrial carrier family**

| Gene name | Protein name | Substrates | 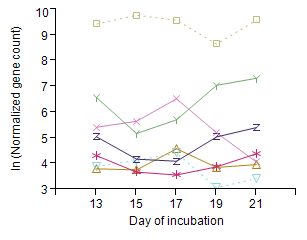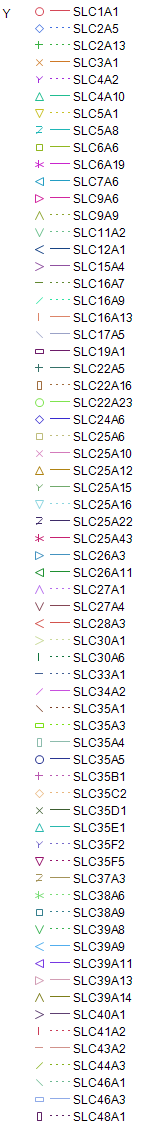 |
| --- | --- | --- | --- |
| [SLC25A1](http://slc.bioparadigms.org/protein?GeneName=SLC25A1) | CIC (citrate carrier) | citrate, isocitrate, malate, PEP |  |
| [SLC25A2](http://slc.bioparadigms.org/protein?GeneName=SLC25A2) | ORC2 (ornithine carrier 2) | ornithine, citrulline, lysine, arginine, histidine |  |
| [SLC25A3](http://slc.bioparadigms.org/protein?GeneName=SLC25A3) | PHC (phosphate carrier) | phosphate |  |
| [SLC25A4](http://slc.bioparadigms.org/protein?GeneName=SLC25A4) | ANT1 (adenine nucleotide translocase-1) | ADP, ATP |  |
| [SLC25A5](http://slc.bioparadigms.org/protein?GeneName=SLC25A5) | ANT2 (adenine nucleotide translocase-2) | ADP, ATP |  |
| [SLC25A5P1](http://slc.bioparadigms.org/protein?GeneName=SLC25A5P1) | pseudogene |  |  |
| [**SLC25A6**](http://slc.bioparadigms.org/protein?GeneName=SLC25A6) | **ANT3 (adenine nucleotide translocase-3)** | **ADP, ATP** |  |
| [SLC25A6P1](http://slc.bioparadigms.org/protein?GeneName=SLC25A6P1) | pseudogene |  |  |
| [SLC25A7](http://slc.bioparadigms.org/protein?GeneName=SLC25A7) | UCP1 (uncoupling protein 1) | H+ |  |
| [SLC25A8](http://slc.bioparadigms.org/protein?GeneName=SLC25A8) | UCP2 (uncoupling protein 2) | H+ |  |
| [SLC25A9](http://slc.bioparadigms.org/protein?GeneName=SLC25A9) | UCP3 (uncoupling protein 3) | H+ |  |
| [**SLC25A10**](http://slc.bioparadigms.org/protein?GeneName=SLC25A10) | **DIC (dicarboxylate carrier)** | **malate, phosphate, succinate, sulphate, thiosulphate** |  |
| [SLC25A11](http://slc.bioparadigms.org/protein?GeneName=SLC25A11) | OGC (oxoglutarate carrier) | 2-oxoglutarate, malate |  |
| [**SLC25A12**](http://slc.bioparadigms.org/protein?GeneName=SLC25A12) | **AGC1 (aspartate / glutamate carrier 1)** | **aspartate, glutamate** |  |
| [SLC25A13](http://slc.bioparadigms.org/protein?GeneName=SLC25A13) | AGC2 (aspartate / glutamate carrier 2) | aspartate, glutamate |  |
| [SLC25A14](http://slc.bioparadigms.org/protein?GeneName=SLC25A14) | UCP5 (uncoupling protein 5) |  |  |
| [**SLC25A15**](http://slc.bioparadigms.org/protein?GeneName=SLC25A15) | **ORC1 (ornithine carrier 1)** | **ornithine, citrulline, lysine, arginine** |  |
| [SLC25A15P1](http://slc.bioparadigms.org/protein?GeneName=SLC25A15P1) | pseudogene |  |  |
| [**SLC25A16**](http://slc.bioparadigms.org/protein?GeneName=SLC25A16) | **GDC (Graves' disease carrier)** |  |  |
| [SLC25A17](http://slc.bioparadigms.org/protein?GeneName=SLC25A17) |  | CoA, FAD, NAD+, AMP, ADP, PAP, dPCoA, FMN |  |
| [SLC25A18](http://slc.bioparadigms.org/protein?GeneName=SLC25A18) | GC2 (glutamate carrier 2) | glutamate |  |
| [SLC25A19](http://slc.bioparadigms.org/protein?GeneName=SLC25A19) | DNC (deoxynucleotide carrier) | thiamine pyrophosphate, thiamine monophosphate, (deoxy)nucleotides |  |
| [SLC25A20](http://slc.bioparadigms.org/protein?GeneName=SLC25A20) | CAC (carnitine / acylcarnitine carrier) | carnitine, acylcarnitine |  |
| [SLC25A20P1](http://slc.bioparadigms.org/protein?GeneName=SLC25A20P1) | pseudogene |  |  |
| [SLC25A21](http://slc.bioparadigms.org/protein?GeneName=SLC25A21) | ODC (oxoadipate carrier) | oxoadipate, oxoglutarate |  |
| [**SLC25A22**](http://slc.bioparadigms.org/protein?GeneName=SLC25A22) | **GC1 (glutamate carrier 1)** | **glutamate** |  |
| [SLC25A23](http://slc.bioparadigms.org/protein?GeneName=SLC25A23) | APC2 | ATP-Mg2+, ATP, ADP, AMP, Pi |  |
| [SLC25A24](http://slc.bioparadigms.org/protein?GeneName=SLC25A24) | APC1 | ATP-Mg2+, ATP, ADP, AMP, Pi |  |
| [SLC25A25](http://slc.bioparadigms.org/protein?GeneName=SLC25A25) | APC3 |  |  |
| [SLC25A26](http://slc.bioparadigms.org/protein?GeneName=SLC25A26) | SAMC | S-adenosyl-methionine, S-adenosyl-homocysteine |  |
| [SLC25A27](http://slc.bioparadigms.org/protein?GeneName=SLC25A27) | UCP4 (uncoupling protein 4) |  |  |
| [SLC25A28](http://slc.bioparadigms.org/protein?GeneName=SLC25A28) | Mitoferrin 2 (Mfrn2) | Fe2+ |  |
| [SLC25A29](http://slc.bioparadigms.org/protein?GeneName=SLC25A29) | ORNT3 | ornithine, acylcarnitine |  |
| [SLC25A30](http://slc.bioparadigms.org/protein?GeneName=SLC25A30) |  |  |  |
| [SLC25A31](http://slc.bioparadigms.org/protein?GeneName=SLC25A31) | AAC4, ANT4 (adenine nucleotide carrier 4) | ADP, ATP |  |
| [SLC25A32](http://slc.bioparadigms.org/protein?GeneName=SLC25A32) | MFT | folate |  |
| [SLC25A33](http://slc.bioparadigms.org/protein?GeneName=SLC25A33) | PNC1 (pyrimidine nucleotide carrier 1) | UTP |  |
| [SLC25A34](http://slc.bioparadigms.org/protein?GeneName=SLC25A34) |  |  |  |
| [SLC25A35](http://slc.bioparadigms.org/protein?GeneName=SLC25A35) |  |  |  |
| [SLC25A36](http://slc.bioparadigms.org/protein?GeneName=SLC25A36) | PNC2 (pyrimidine nucleotide carrier 2) | pyrimidine nucleotides |  |
| [SLC25A37](http://slc.bioparadigms.org/protein?GeneName=SLC25A37) | Mitoferrin 1 (Mfrn1) | Fe2+ |  |
| [SLC25A38](http://slc.bioparadigms.org/protein?GeneName=SLC25A38) |  | glycine ? |  |
| [SLC25A39](http://slc.bioparadigms.org/protein?GeneName=SLC25A39) |  |  |  |
| [SLC25A40](http://slc.bioparadigms.org/protein?GeneName=SLC25A40) |  |  |  |
| [SLC25A41](http://slc.bioparadigms.org/protein?GeneName=SLC25A41) | APC4 | ATP-Mg / Pi |  |
| [SLC25A42](http://slc.bioparadigms.org/protein?GeneName=SLC25A42) |  | CoA, ADP, ATP, adenosine 3',5'-diphosphate, dPCoA |  |
| [**SLC25A43**](http://slc.bioparadigms.org/protein?GeneName=SLC25A43) |  |  |  |
| [SLC25A44](http://slc.bioparadigms.org/protein?GeneName=SLC25A44) |  |  |  |
| [SLC25A45](http://slc.bioparadigms.org/protein?GeneName=SLC25A45) |  |  |  |
| [SLC25A46](http://slc.bioparadigms.org/protein?GeneName=SLC25A46) |  |  |  |
| [SLC25A47](http://slc.bioparadigms.org/protein?GeneName=SLC25A47) |  |  |  |
| [SLC25A48](http://slc.bioparadigms.org/protein?GeneName=SLC25A48) |  |  |  |
| [SLC25A49](http://slc.bioparadigms.org/protein?GeneName=SLC25A49) | MTCH1 |  |  |
| [SLC25A50](http://slc.bioparadigms.org/protein?GeneName=SLC25A50) | MTCH2 |  |  |
| [SLC25A51](http://slc.bioparadigms.org/protein?GeneName=SLC25A51) | MCART1 |  |  |
| [SLC25A51P1](http://slc.bioparadigms.org/protein?GeneName=SLC25A51P1) | pseudogene |  |  |
| [SLC25A51P2](http://slc.bioparadigms.org/protein?GeneName=SLC25A51P2) | pseudogene |  |  |
| [SLC25A51P3](http://slc.bioparadigms.org/protein?GeneName=SLC25A51P3) | pseudogene |  |  |
| [SLC25A52](http://slc.bioparadigms.org/protein?GeneName=SLC25A52) | MCART2 |  |  |
| [SLC25A53](http://slc.bioparadigms.org/protein?GeneName=SLC25A53) | MCART6 |  |  |

**SLC26 Multifunctional anion exchanger family**

| Gene name | Protein name | Substrates | 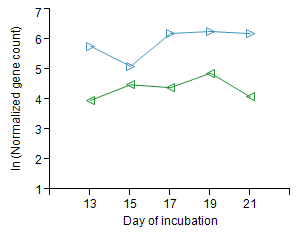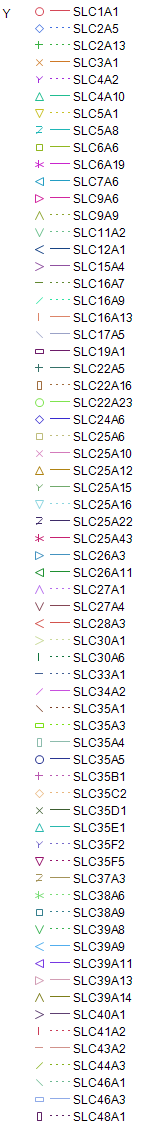 |
| --- | --- | --- | --- |
| [SLC26A1](http://slc.bioparadigms.org/protein?GeneName=SLC26A1) | SAT1, SLC26A1 | SO42-, oxalate, glyoxylate |  |
| [SLC26A2](http://slc.bioparadigms.org/protein?GeneName=SLC26A2) | DTDST, SLC26A2 | SO42-, oxalate, Cl- |  |
| [**SLC26A3**](http://slc.bioparadigms.org/protein?GeneName=SLC26A3) | **DRA, CLD, SLC26A3** | **Cl-, HCO3-, oxalate** |  |
| [SLC26A4](http://slc.bioparadigms.org/protein?GeneName=SLC26A4) | PDS, Pendrin, SLC26A4 | I-, Cl-, HCO3- |  |
| [SLC26A5](http://slc.bioparadigms.org/protein?GeneName=SLC26A5) | Prestin, SLC26A5 | Cl-, formate, oxalate, SO42- |  |
| [SLC26A6](http://slc.bioparadigms.org/protein?GeneName=SLC26A6) | CFEX, PAT1, SLC26A6 | Cl-, HCO3-, oxalate, OH-, formate |  |
| [SLC26A7](http://slc.bioparadigms.org/protein?GeneName=SLC26A7) | SUT2, SLC26A7 | Cl-, HCO3-, OH-, SO42-, Ch: Cl- |  |
| [SLC26A8](http://slc.bioparadigms.org/protein?GeneName=SLC26A8) | TAT1, SLC26A8 | Cl-, HCO3-, OH- |  |
| [SLC26A9](http://slc.bioparadigms.org/protein?GeneName=SLC26A9) | SLC26A9 | Cl-, HCO3, Ch: Cl-, HCO3- |  |
| [SLC26A10](http://slc.bioparadigms.org/protein?GeneName=SLC26A10) | (SLC26A10) |  |  |
| [**SLC26A11**](http://slc.bioparadigms.org/protein?GeneName=SLC26A11) | **SUT1, KBAT, SLC26A11** | **Cl-, HCO3-, SO42-, oxalate (?), Ch: Cl-** |  |

**SLC27 Fatty acid transporter family**

| Gene name | Protein name | Substrates | 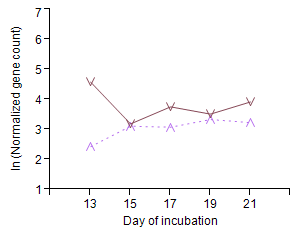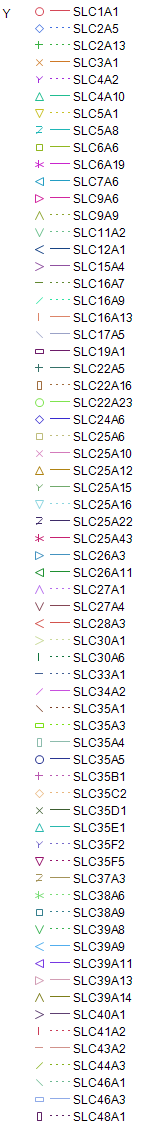 |
| --- | --- | --- | --- |
| [**SLC27A1**](http://slc.bioparadigms.org/protein?GeneName=SLC27A1) | **FATP1** | **LCFA, VLCFA** |  |
| [SLC27A2](http://slc.bioparadigms.org/protein?GeneName=SLC27A2) | FATP2 | LCFA, VLCFA |  |
| [SLC27A3](http://slc.bioparadigms.org/protein?GeneName=SLC27A3) | FATP3 | LCFA, VLCFA |  |
| [**SLC27A4**](http://slc.bioparadigms.org/protein?GeneName=SLC27A4) | **FATP4** | **LCFA, VLCFA** |  |
| [SLC27A5](http://slc.bioparadigms.org/protein?GeneName=SLC27A5) | FATP5 | LCFA, bile acids |  |
| [SLC27A6](http://slc.bioparadigms.org/protein?GeneName=SLC27A6) | FATP6 | LCFA, VLCFA |  |

**SLC28 Na+-coupled nucleoside transport family**

| Gene name | Protein name | Substrates | 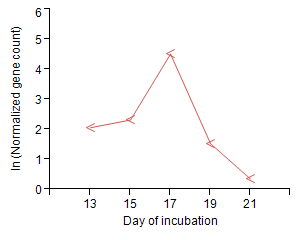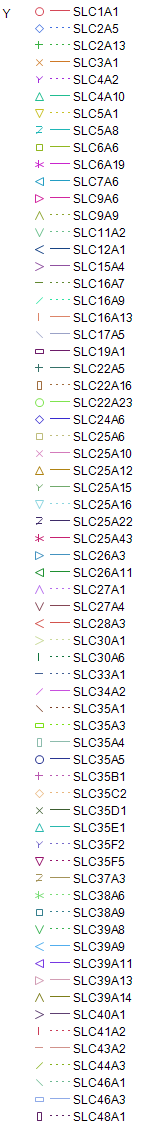 |
| --- | --- | --- | --- |
| [SLC28A1](http://slc.bioparadigms.org/protein?GeneName=SLC28A1) | CNT1 |  |  |
| [SLC28A2](http://slc.bioparadigms.org/protein?GeneName=SLC28A2) | CNT2 |  |  |
| [**SLC28A3**](http://slc.bioparadigms.org/protein?GeneName=SLC28A3) | **CNT3** |  |  |

**SLC29 Facilitative nucleoside transporter family**

| Gene name | Protein name | Substrates |
| --- | --- | --- |
| [SLC29A1](http://slc.bioparadigms.org/protein?GeneName=SLC29A1) | ENT1 |  |
| [SLC29A2](http://slc.bioparadigms.org/protein?GeneName=SLC29A2) | ENT2 |  |
| [SLC29A3](http://slc.bioparadigms.org/protein?GeneName=SLC29A3) | ENT3 |  |
| [SLC29A4](http://slc.bioparadigms.org/protein?GeneName=SLC29A4) | ENT4 |  |

**SLC30 Zinc efflux family**

| Gene name | Protein name | Substrates | 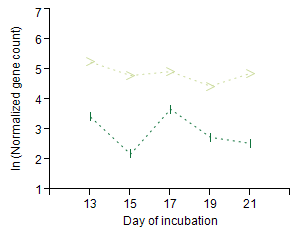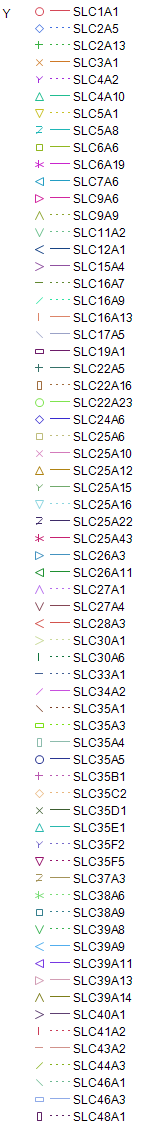 |
| --- | --- | --- | --- |
| [**SLC30A1**](http://slc.bioparadigms.org/protein?GeneName=SLC30A1) |  |  |  |
| [SLC30A2](http://slc.bioparadigms.org/protein?GeneName=SLC30A2) |  |  |  |
| [SLC30A3](http://slc.bioparadigms.org/protein?GeneName=SLC30A3) |  |  |  |
| [SLC30A4](http://slc.bioparadigms.org/protein?GeneName=SLC30A4) |  |  |  |
| [SLC30A5](http://slc.bioparadigms.org/protein?GeneName=SLC30A5) |  |  |  |
| [**SLC30A6**](http://slc.bioparadigms.org/protein?GeneName=SLC30A6) |  |  |  |
| [SLC30A7](http://slc.bioparadigms.org/protein?GeneName=SLC30A7) |  |  |  |
| [SLC30A8](http://slc.bioparadigms.org/protein?GeneName=SLC30A8) |  |  |  |
| [SLC30A9](http://slc.bioparadigms.org/protein?GeneName=SLC30A9) |  |  |  |
| [SLC30A10](http://slc.bioparadigms.org/protein?GeneName=SLC30A10) |  |  |  |

**SLC31 Copper transporter family**

| Gene name | Protein name | Substrates |
| --- | --- | --- |
| [SLC31A1](http://slc.bioparadigms.org/protein?GeneName=SLC31A1) | CTR1 | copper (I), cisplatin |
| [SLC31A1P1](http://slc.bioparadigms.org/protein?GeneName=SLC31A1P1) | pseudogene |  |
| [SLC31A2](http://slc.bioparadigms.org/protein?GeneName=SLC31A2) | CTR2 | copper, cisplatin |

**SLC32 Vesicular inhibitory amino acid transporter family**

| Gene name | Protein name | Substrates |
| --- | --- | --- |
| [SLC32A1](http://slc.bioparadigms.org/protein?GeneName=SLC32A1) | VIAAT | GABA / glycine |

**SLC33 Acetyl-CoA transporter family**

| Gene name | Protein name | Substrates | 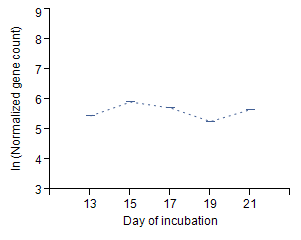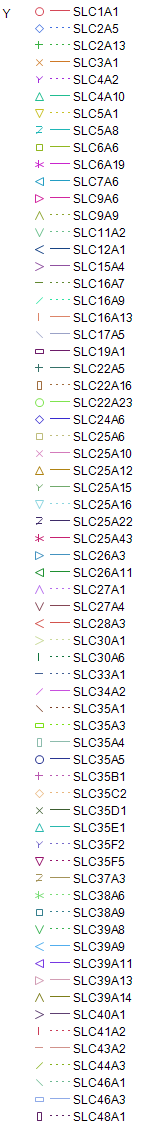 |
| --- | --- | --- | --- |
| [**SLC33A1**](http://slc.bioparadigms.org/protein?GeneName=SLC33A1) | **ACATN1** | **acetyl-CoA** |  |

**SLC34 Type II Na+-phosphate cotransporter family**

| Gene name | Protein name | Substrates | 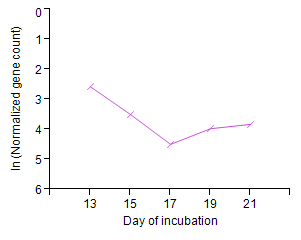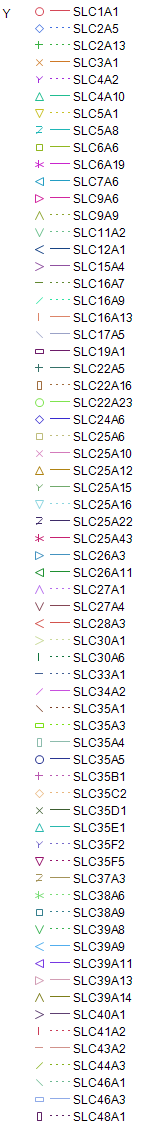 |
| --- | --- | --- | --- |
| [SLC34A1](http://slc.bioparadigms.org/protein?GeneName=SLC34A1) | NaPi-IIa | inorganic phosphate (divalent) |  |
| [**SLC34A2**](http://slc.bioparadigms.org/protein?GeneName=SLC34A2) | **NaPi-IIb** | **inorganic phosphate (divalent)** |  |
| [SLC34A3](http://slc.bioparadigms.org/protein?GeneName=SLC34A3) | NaPi-IIc | inorganic phosphate (divalent) |  |

**SLC35 Nucleoside-sugar transporter family**

| Gene name | Protein name | Substrates | 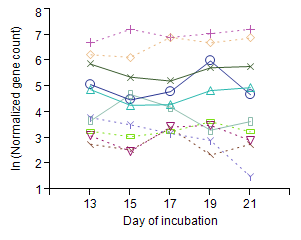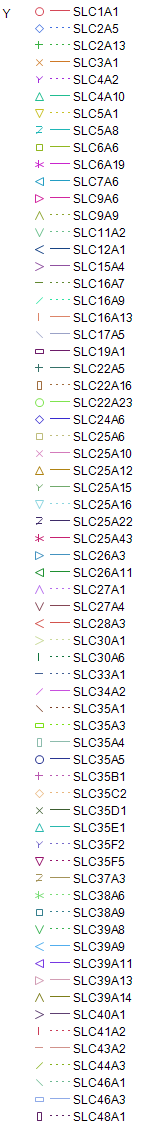 |
| --- | --- | --- | --- |
| [**SLC35A1**](http://slc.bioparadigms.org/protein?GeneName=SLC35A1) | **CST** | **CMP-sialic acid** |  |
| [SLC35A2](http://slc.bioparadigms.org/protein?GeneName=SLC35A2) | UGT | UDP-galactose, UDP-N-acetylgalactosamine |  |
| [**SLC35A3**](http://slc.bioparadigms.org/protein?GeneName=SLC35A3) |  | **UDP-N-acetylglucosamine** |  |
| [**SLC35A4**](http://slc.bioparadigms.org/protein?GeneName=SLC35A4) |  |  |  |
| [**SLC35A5**](http://slc.bioparadigms.org/protein?GeneName=SLC35A5) |  |  |  |
| [**SLC35B1**](http://slc.bioparadigms.org/protein?GeneName=SLC35B1) |  |  |  |
| [SLC35B2](http://slc.bioparadigms.org/protein?GeneName=SLC35B2) | PAPST1 | PAPS |  |
| [SLC35B3](http://slc.bioparadigms.org/protein?GeneName=SLC35B3) | PAPST2 | PAPS |  |
| [SLC35B4](http://slc.bioparadigms.org/protein?GeneName=SLC35B4) | YEA | UDP-xylose, UDP-N-acetylglucosamine |  |
| [SLC35C1](http://slc.bioparadigms.org/protein?GeneName=SLC35C1) | FUCT1 | GDP-fucose |  |
| [**SLC35C2**](http://slc.bioparadigms.org/protein?GeneName=SLC35C2) | OVCOV1 | GDP-fucose (?) |  |
| [**SLC35D1**](http://slc.bioparadigms.org/protein?GeneName=SLC35D1) | UGTREL7 | UDP-glucuronic acid, UDP-N-acetylgalactosamine |  |
| [SLC35D2](http://slc.bioparadigms.org/protein?GeneName=SLC35D2) | HFRC1 | UDP-N-acetylglucosamine |  |
| [SLC35D3](http://slc.bioparadigms.org/protein?GeneName=SLC35D3) | FRCL1 |  |  |
| [**SLC35E1**](http://slc.bioparadigms.org/protein?GeneName=SLC35E1) |  |  |  |
| [SLC35E2](http://slc.bioparadigms.org/protein?GeneName=SLC35E2) |  |  |  |
| [SLC35E3](http://slc.bioparadigms.org/protein?GeneName=SLC35E3) |  |  |  |
| [SLC35E4](http://slc.bioparadigms.org/protein?GeneName=SLC35E4) |  |  |  |
| [SLC35F1](http://slc.bioparadigms.org/protein?GeneName=SLC35F1) |  |  |  |
| [**SLC35F2**](http://slc.bioparadigms.org/protein?GeneName=SLC35F2) |  |  |  |
| [SLC35F3](http://slc.bioparadigms.org/protein?GeneName=SLC35F3) |  |  |  |
| [SLC35F4](http://slc.bioparadigms.org/protein?GeneName=SLC35F4) |  |  |  |
| [**SLC35F5**](http://slc.bioparadigms.org/protein?GeneName=SLC35F5) |  |  |  |
| [SLC35F6](http://slc.bioparadigms.org/protein?GeneName=SLC35F6) | C2orf18 |  |  |
| [SLC35G1](http://slc.bioparadigms.org/protein?GeneName=SLC35G1) |  |  |  |
| [SLC35G2](http://slc.bioparadigms.org/protein?GeneName=SLC35G2) |  |  |  |
| [SLC35G3](http://slc.bioparadigms.org/protein?GeneName=SLC35G3) |  |  |  |
| [SLC35G4](http://slc.bioparadigms.org/protein?GeneName=SLC35G4) |  |  |  |
| [SLC35G5](http://slc.bioparadigms.org/protein?GeneName=SLC35G5) |  |  |  |
| [SLC35G6](http://slc.bioparadigms.org/protein?GeneName=SLC35G6) |  |  |  |

**SLC36 Proton-coupled amino acid transporter family**

| Gene name | Protein name | Substrates |
| --- | --- | --- |
| [SLC36A1](http://slc.bioparadigms.org/protein?GeneName=SLC36A1) | PAT1 | GABA, P, G, beta-alanine |
| [SLC36A2](http://slc.bioparadigms.org/protein?GeneName=SLC36A2) | PAT2 | P, G, A, hydroxyproline |
| [SLC36A3](http://slc.bioparadigms.org/protein?GeneName=SLC36A3) | PAT3 |  |
| [SLC36A4](http://slc.bioparadigms.org/protein?GeneName=SLC36A4) | PAT4 | P, tryptophan |

**SLC37 Sugar-phosphate/phosphate exchanger family**

| Gene name | Protein name | Substrates | 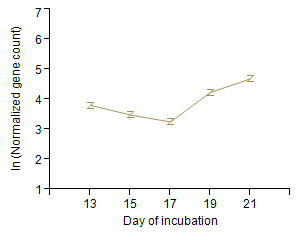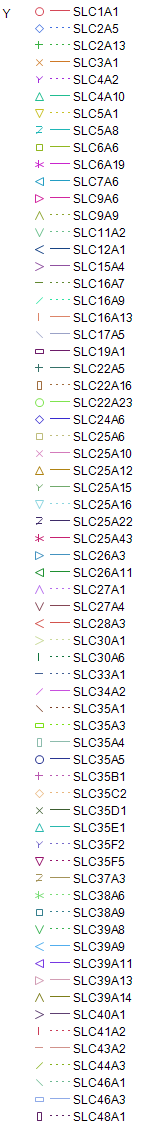 |
| --- | --- | --- | --- |
| [SLC37A1](http://slc.bioparadigms.org/protein?GeneName=SLC37A1) | SLC37A1, SPX1 |  |  |
| [SLC37A2](http://slc.bioparadigms.org/protein?GeneName=SLC37A2) | SLC37A2, SPX2 |  |  |
| [**SLC37A3**](http://slc.bioparadigms.org/protein?GeneName=SLC37A3) | **SLC37A3, SPX3** |  |  |
| [SLC37A4](http://slc.bioparadigms.org/protein?GeneName=SLC37A4) | SLC37A4, G6PT, SPX4 |  |  |

**SLC38 System A and System N sodium-coupled neutral amino acid transporter family**

| Gene name | Protein name | Substrates | 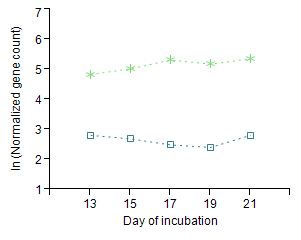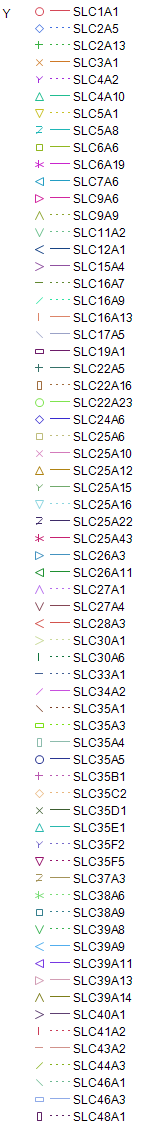 |
| --- | --- | --- | --- |
| [SLC38A1](http://slc.bioparadigms.org/protein?GeneName=SLC38A1) | SNAT1 | Q, A, N, C, H, S |  |
| [SLC38A2](http://slc.bioparadigms.org/protein?GeneName=SLC38A2) | SNAT2 | A, N, C, Q, G, H, M, P, S |  |
| [SLC38A3](http://slc.bioparadigms.org/protein?GeneName=SLC38A3) | SNAT3 | Q, H, A, N |  |
| [SLC38A4](http://slc.bioparadigms.org/protein?GeneName=SLC38A4) | SNAT4 | A, N, C, G, S, T |  |
| [SLC38A5](http://slc.bioparadigms.org/protein?GeneName=SLC38A5) | SNAT5 | Q, N, H, S |  |
| [**SLC38A6**](http://slc.bioparadigms.org/protein?GeneName=SLC38A6) | **SNAT6** |  |  |
| [SLC38A7](http://slc.bioparadigms.org/protein?GeneName=SLC38A7) | SNAT7 | Q, H, S ,A, N |  |
| [SLC38A8](http://slc.bioparadigms.org/protein?GeneName=SLC38A8) | N/A |  |  |
| [**SLC38A9**](http://slc.bioparadigms.org/protein?GeneName=SLC38A9) | **N/A** |  |  |
| [SLC38A10](http://slc.bioparadigms.org/protein?GeneName=SLC38A10) | N/A |  |  |
| [SLC38A11](http://slc.bioparadigms.org/protein?GeneName=SLC38A11) | N/A |  |  |

**SLC39 Metal ion transporter family**

| Gene name | Protein name | Substrates | 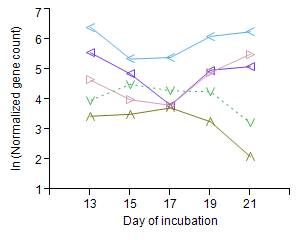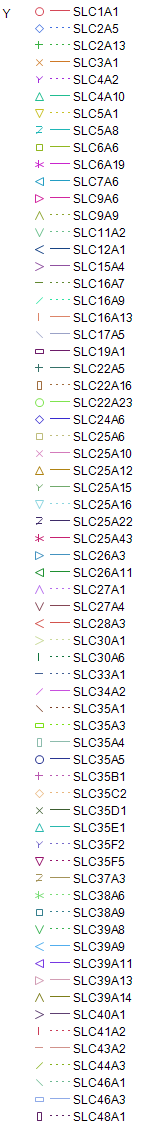 |
| --- | --- | --- | --- |
| [SLC39A1](http://slc.bioparadigms.org/protein?GeneName=SLC39A1) | ZIP1, ZIRTL | Zn |  |
| [SLC39A2](http://slc.bioparadigms.org/protein?GeneName=SLC39A2) | ZIP2, Eti-1, 6A1 | Zn |  |
| [SLC39A3](http://slc.bioparadigms.org/protein?GeneName=SLC39A3) | ZIP3 | Zn, not specific |  |
| [SLC39A4](http://slc.bioparadigms.org/protein?GeneName=SLC39A4) | ZIP4 | Zn |  |
| [SLC39A5](http://slc.bioparadigms.org/protein?GeneName=SLC39A5) | ZIP5, LZT-Hs7 | Zn |  |
| [SLC39A6](http://slc.bioparadigms.org/protein?GeneName=SLC39A6) | ZIP6, LIV-1 | Zn |  |
| [SLC39A7](http://slc.bioparadigms.org/protein?GeneName=SLC39A7) | ZIP7, HKE4, RING5 | Zn, Mn |  |
| [**SLC39A8**](http://slc.bioparadigms.org/protein?GeneName=SLC39A8) | **ZIP8, BIGM103, LZT-Hs6** | **Zn, Cd, Mn** |  |
| [**SLC39A9**](http://slc.bioparadigms.org/protein?GeneName=SLC39A9) | **ZIP9** |  |  |
| [SLC39A10](http://slc.bioparadigms.org/protein?GeneName=SLC39A10) | ZIP10, LZT-Hs2 | Zn |  |
| [**SLC39A11**](http://slc.bioparadigms.org/protein?GeneName=SLC39A11) | **ZIP11** |  |  |
| [SLC39A12](http://slc.bioparadigms.org/protein?GeneName=SLC39A12) | ZIP12j, LZT-Hs8 | Zn |  |
| [**SLC39A13**](http://slc.bioparadigms.org/protein?GeneName=SLC39A13) | **ZIP13, LZT-Hs9** | **Zn** |  |
| [**SLC39A14**](http://slc.bioparadigms.org/protein?GeneName=SLC39A14) | **ZIP14, LZT-Hs4** | **Zn, Fe, Mn, Cd** |  |

**SLC40 Basolateral iron transporter family**

| Gene name | Protein name | Substrates | 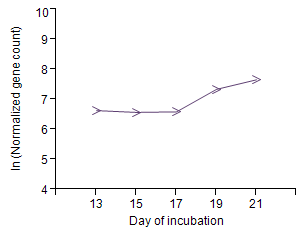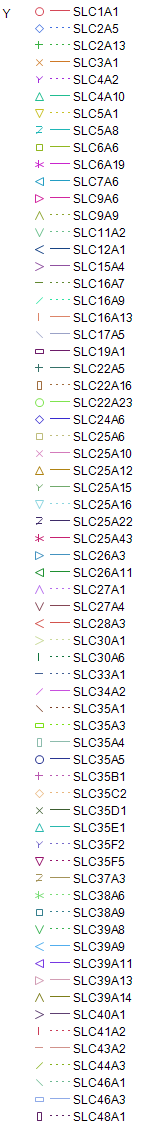 |
| --- | --- | --- | --- |
| [**SLC40A1**](http://slc.bioparadigms.org/protein?GeneName=SLC40A1) | **ferroportin (FPN1)** | **ferrous iron** |  |

**SLC41 MgtE-like magnesium transporter family**

| Gene name | Protein name | Substrates | 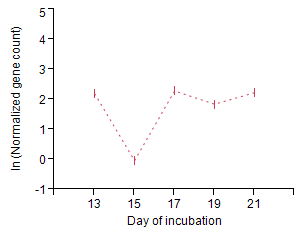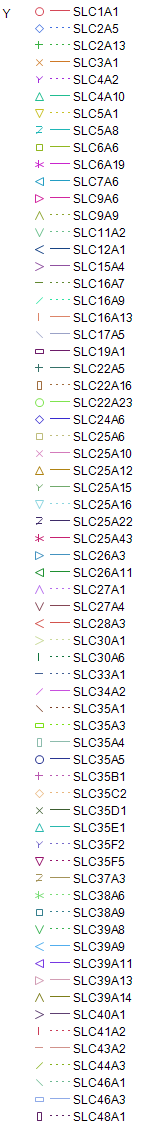 |
| --- | --- | --- | --- |
| [SLC41A1](http://slc.bioparadigms.org/protein?GeneName=SLC41A1) | MgtE | Mg2+ (Sr2+, Zn2+, Cu2+, Fe2+, Co2+, Ba2+, Cd2+) |  |
| [**SLC41A2**](http://slc.bioparadigms.org/protein?GeneName=SLC41A2) |  | **Mg2+ (Ba2+, Ni2+, Co2+, Fe2+, Mn2+)** |  |
| [SLC41A3](http://slc.bioparadigms.org/protein?GeneName=SLC41A3) |  |  |  |

**SLC42 Rh ammonium transporter family**

| Gene name | Protein name | Substrates |
| --- | --- | --- |
| [SLC42A1](http://slc.bioparadigms.org/protein?GeneName=SLC42A1) | RhAG | NH4+, NH3 |
| [SLC42A2](http://slc.bioparadigms.org/protein?GeneName=SLC42A2) | RhBG | NH4+, NH3, methyl amine, methyl ammonium |
| [SLC42A3](http://slc.bioparadigms.org/protein?GeneName=SLC42A3) | RhCG | NH4+, NH3 |

**SLC43 Na+-independent, system-L-like amino acid transporter family**

| Gene name | Protein name | Substrates | 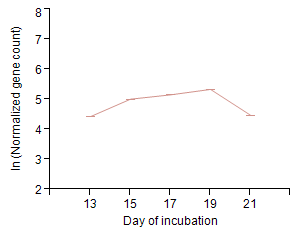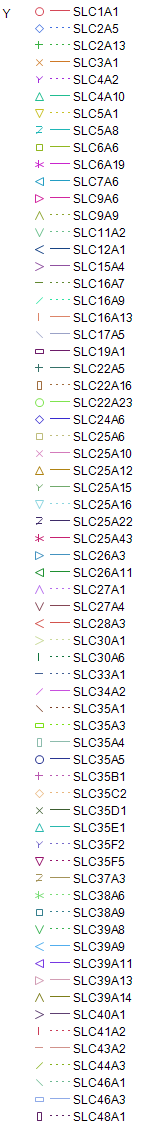 |
| --- | --- | --- | --- |
| [SLC43A1](http://slc.bioparadigms.org/protein?GeneName=SLC43A1) | LAT3 | L-BCAAs, amino alcohols |  |
| [**SLC43A2**](http://slc.bioparadigms.org/protein?GeneName=SLC43A2) | **LAT4** | **L-BCAAs, amino alcohols** |  |
| [SLC43A3](http://slc.bioparadigms.org/protein?GeneName=SLC43A3) | EEG1 |  |  |

**SLC44 Choline-like transporter family**

| Gene name | Protein name | Substrates | 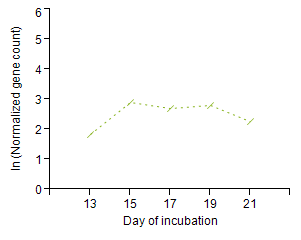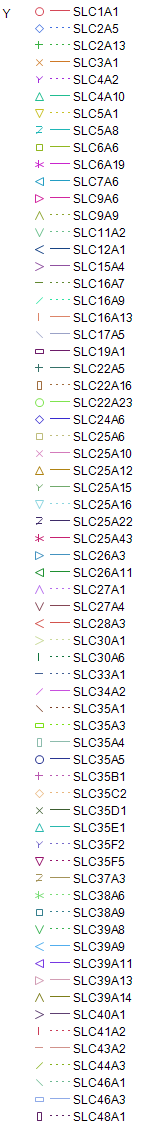 |
| --- | --- | --- | --- |
| [SLC44A1](http://slc.bioparadigms.org/protein?GeneName=SLC44A1) | CTL1 | choline |  |
| [SLC44A2](http://slc.bioparadigms.org/protein?GeneName=SLC44A2) | CTL2 | choline |  |
| [**SLC44A3**](http://slc.bioparadigms.org/protein?GeneName=SLC44A3) | **CTL3** |  |  |
| [SLC44A4](http://slc.bioparadigms.org/protein?GeneName=SLC44A4) | CTL4 |  |  |
| [SLC44A5](http://slc.bioparadigms.org/protein?GeneName=SLC44A5) | CTL5 |  |  |

**SLC45 Putative sugar transporter family**

| Gene name | Protein name | Substrates |
| --- | --- | --- |
| [SLC45A1](http://slc.bioparadigms.org/protein?GeneName=SLC45A1) | Past-A, DNB5 | glucose, galactose |
| [SLC45A2](http://slc.bioparadigms.org/protein?GeneName=SLC45A2) | MATP, AIM1, underwhite |  |
| [SLC45A3](http://slc.bioparadigms.org/protein?GeneName=SLC45A3) | Prostein |  |
| [SLC45A4](http://slc.bioparadigms.org/protein?GeneName=SLC45A4) |  |  |

**SLC46 Folate transporter family**

| Gene name | Protein name | Substrates | 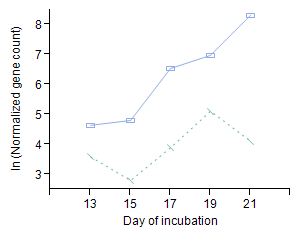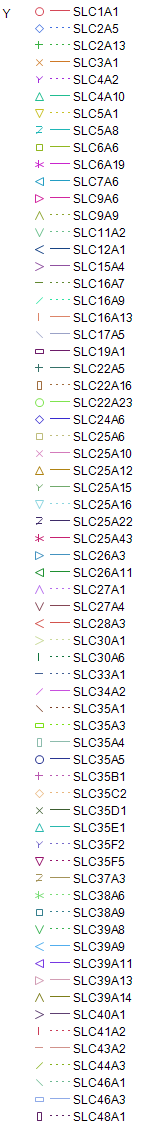 |
| --- | --- | --- | --- |
| [**SLC46A1**](http://slc.bioparadigms.org/protein?GeneName=SLC46A1) | **PCFT** | **folates, antifolates** |  |
| [SLC46A2](http://slc.bioparadigms.org/protein?GeneName=SLC46A2) | TSCOT | unknown |  |
| [**SLC46A3**](http://slc.bioparadigms.org/protein?GeneName=SLC46A3) |  | **unknown** |  |

**SLC47 Multidrug and Toxin Extrusion (MATE) family**

| Gene name | Protein name | Substrates |
| --- | --- | --- |
| [SLC47A1](http://slc.bioparadigms.org/protein?GeneName=SLC47A1) |  | tetraethylammonium (TEA), 1-methyl-4-phenylpyridinium (MPP), cimetidine, metformin, guanidine, procainamide, cephalexin, cephradine |
| [SLC47A2](http://slc.bioparadigms.org/protein?GeneName=SLC47A2) |  | TEA, MPP, cimetidine, metformin, guanidine, procainamide |

**SLC48 Heme transporter family**

| Gene name | Protein name | Substrates | 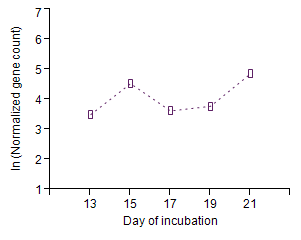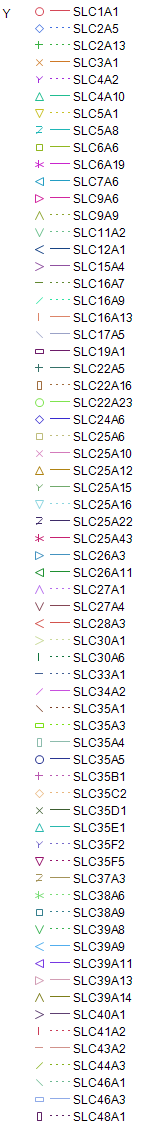 |
| --- | --- | --- | --- |
| [**SLC48A1**](http://slc.bioparadigms.org/protein?GeneName=SLC48A1) | **HRG-1** | **heme** |  |

**SLC49 FLVCR-related transporter family**

| Gene name | Protein name | Substrates |
| --- | --- | --- |
| [SLC49A1](http://slc.bioparadigms.org/protein?GeneName=SLC49A1) | FLVCR1 | heme |
| [SLC49A2](http://slc.bioparadigms.org/protein?GeneName=SLC49A2) | FLVCR2 | heme |
| [SLC49A3](http://slc.bioparadigms.org/protein?GeneName=SLC49A3) | MFSD7 | unknown |
| [SLC49A4](http://slc.bioparadigms.org/protein?GeneName=SLC49A4) | DIRC2 | unknown |

**SLC50 Sugar efflux transporters**

| Gene name | Protein name | Substrates |
| --- | --- | --- |
| [SLC50A1](http://slc.bioparadigms.org/protein?GeneName=SLC50A1) | RAG1AP | glucose |

**SLC51 Transporters of steroid-derived molecules**

| Gene name | Protein name | Substrates |
| --- | --- | --- |
| [SLC51A](http://slc.bioparadigms.org/protein?GeneName=SLC51A) | OST alpha | bile acids |
| [SLC51B](http://slc.bioparadigms.org/protein?GeneName=SLC51B) | OST beta | steroids |

**SLC52 Riboflavin transporter family RFVT/SLC52**

| Gene name |
| --- |
| [SLC52A1](http://slc.bioparadigms.org/protein?GeneName=SLC52A1) |
| [SLC52A2](http://slc.bioparadigms.org/protein?GeneName=SLC52A2) |
| [SLC52A3](http://slc.bioparadigms.org/protein?GeneName=SLC52A3) |
