# Supplementary material for: Temporal transcriptome analysis of the chicken embryo yolk sac
Source: BMC Genomics. 2014 Aug 19;15(1):690. doi: 10.1186/1471-2164-15-690 (PMC4246430; doi:10.1186/1471-2164-15-690)
Supplement: Supplementary file 10 — Additional file 10: YS Hemoglobin catabolism and bile biosynthesis. (DOCX 640 KB) [file 12864_2014_6680_MOESM10_ESM.docx]

A remarkable change in the color of the yolk occurs in the last days of incubation. The yolk changes its color from yellow to green. We found that the origin of these bile acids is not the intestinal gallbladder bile (the intestine in the lower figure (A) is empty of bile on E17, whereas the yolk is green), the origin is the YSwhere genes involved in bile synthesis and conjugation increased during incubation.

| 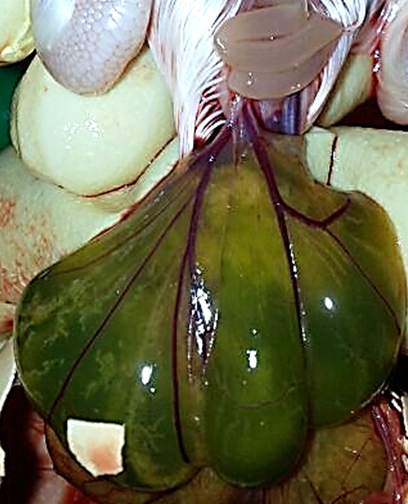  A  **Intestine**  **YS** | |
| --- | --- |
| 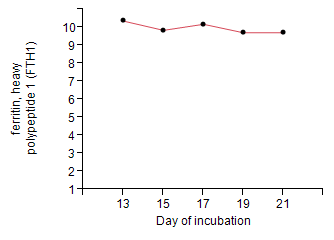  C | 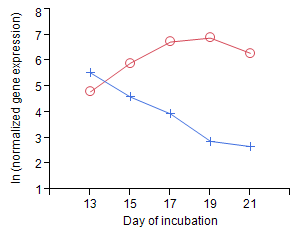  B |
|  | 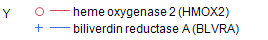 |
| 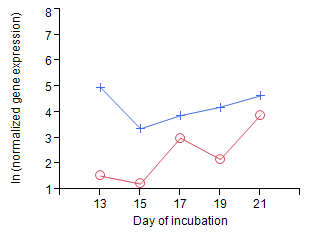  D | |
| 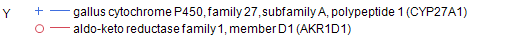 | |
